# Proteomic Characterization of Middle Ear Fluid Confirms Neutrophil Extracellular Traps as a Predominant Innate Immune Response in Chronic Otitis Media

**DOI:** 10.1371/journal.pone.0152865

**Published:** 2016-04-14

**Authors:** Stephanie Val, Marian Poley, Kristy Brown, Rachel Choi, Stephanie Jeong, Annie Colberg-Poley, Mary C. Rose, Karuna C. Panchapakesan, Joe C. Devaney, Marcos Perez-Losada, Diego Preciado

**Affiliations:** 1 Sheikh Zayed Center for Pediatric Surgical Innovation, Children’s National Health System, Washington, DC, United States of America; 2 Center for Genetic Medicine Research, Children’s National Health System, Washington, DC, United States of America; 3 Division of Pediatric Otolaryngology, Children’s National Health System, Washington, DC, United States of America; The Hospital for Sick Children and The University of Toronto, CANADA

## Abstract

**Background:**

Chronic Otitis Media (COM) is characterized by middle ear effusion (MEE) and conductive hearing loss. MEE reflect mucus hypersecretion, but global proteomic profiling of the mucosal components are limited.

**Objective:**

This study aimed at characterizing the proteome of MEEs from children with COM with the goal of elucidating important innate immune responses.

**Method:**

MEEs were collected from children (n = 49) with COM undergoing myringotomy. Mass spectrometry was employed for proteomic profiling in nine samples. Independent samples were further analyzed by cytokine multiplex assay, immunoblotting, neutrophil elastase activity, next generation DNA sequencing, and/or immunofluorescence analysis.

**Results:**

109 unique and common proteins were identified by MS. A majority were innate immune molecules, along with typically intracellular proteins such as histones and actin. 19.5% percent of all mapped peptide counts were from proteins known to be released by neutrophils. Immunofluorescence and immunoblotting demonstrated the presence of neutrophil extracellular traps (NETs) in every MEE, along with MUC5B colocalization. DNA found in effusions revealed unfragmented DNA of human origin.

**Conclusion:**

Proteomic analysis of MEEs revealed a predominantly neutrophilic innate mucosal response in which MUC5B is associated with NET DNA. NETs are a primary macromolecular constituent of human COM middle ear effusions.

## Introduction

Otitis Media (OM) is one of the most common conditions of early childhood accounting for a very high proportion of all pediatric office visits and surgeries annually[1,2] at a national health care cost estimated to be greater than $1 billion[3,4]. Chronic Otitis Media (COM) typically results as a long term sequelae of recurrent acute middle ear infections, and is characterized by persistence of middle ear effusion (MEE), most frequently mucoid[5,6,7]. This viscous middle ear effusion has been classically described as ‘glue ear’ [8] and is associated with conductive hearing loss, effusion non-clearance, and increased likelihood of requiring surgical tympanostomy tube placement [9,10,11,12,13]. Our group performed proteomic analysis of mucoid middle ear effusions from children with COM and reported that mucin glycoprotein MUC5B is the predominant mucin [14]. However, a detailed global proteomic analysis to identify and validation of innate immune proteins that are functionally important in mucosal immunity in MEE has not been performed by us or others.

Little is known about the biological mechanisms in OM that fully explain the progression in OM from acute OM (AOM) to COM. It has been well-demonstrated that during this process, the healthy single layered middle ear epithelium remodels into a pseudo stratified epithelium in COM able to potently produce mucins[15,16,17]. This process has long been thought to be influenced by pro-inflammatory mediators, specifically through bacterial activation of epithelial proinflammatory pathways (reviewed in [18]). Some have attributed the progression from AOM to COM as an ‘allergic’ response characterized by presence of eosin eosinophilic markers and mediators in middle ear effusion (MEE)[19]. Others have argued that COM represents more of a neutrophilic mediated response[20].

Recently, separate studies of MEE from children with either recurrent AOM or COMdemonstrated the presence of neutrophil extracellular traps (NETs) in the middle ear fluid and showed that the extensive DNA stranding evident within the MEE is largely NET derived [21,22] NETs are a relative recently discovered innate immunity mechanism by which neutrophils are able to kill pathogens[23]. During “NETosis” neutrophils recognize bacterial components or pro-inflammatory cytokines and undergo a cell death event, whereby they release their DNA along with a variety of bactericidal peptides which are then able to trap and kill pathogens. The DNA stranding characteristic of NETs in some instances can also integrate as a host component of bacterial biofilms, paradoxically allowing for some pathogens to elude immune detection[24]. Indeed, COM has been proposed to represent a condition which persists primarily due to the presence of bacterial biofilms on middle ear mucosa [25,26].

For this study we posited that, an unbiased proteomics approach would reveal neutrophilic markers and NETs would be abundant in COM fluid. We aimed to demonstrate that NETs were present in the MEE of children with COM and characterize the COM MEE NET and proteome focusing the analysis on mediators associated with innate immune responses.

## Methods

### Sample Collection and Preparation

The Institutional Review Board Committee of Children’s National Health System approved this study. After obtaining written informed consent from the legal guardian, effusions from children aged 0–35 months with COM, undergoing myringotomy with tube placement irrespective of race/ethnicity/gender were collected. For sample analysis, effusions from each ear were pooled into one sample per patient. Exclusion criteria included: cleft palate or other craniofacial dysmorphic syndromes, immunosuppressive states or conditions, cystic fibrosis, immotile cilia syndrome, or prior history of skull base radiation therapy or skull base malignancy. A total of 49 samples of MEE from x pediatric patients with COM were collected fresh, aliqouted, and if not immediately used for experiments, frozen at -80°C as previously described[14]. Importantly, in order to get samples into suspension, these were first homogenized in 1ml of sterile phosphate buffered saline (PBS), assisted by the use of an 18g syringe if needed due to sample viscosity (sample passed in needle 10 times—helping break up the chunks). of MEE were used for proteomic, cytokine, Western blot, immunofluorescence, and DNA analyses.

### Proteomic Analyses

Protein concentration was determined from MEE aliquots using Bicinchoninic Acid Microtiter Plate Assays (Pierce, Rockland, Illinois). As some of the effusion samples had blood contamination, a serum sample was also collected as a control for protein presence for the proteomic analysis.

Proteins were separated by SDS-PAGE electrophoresis and analyzed by proteomics as previously described[14]. Briefly, 100μg of effusion sample protein (n = 9) was dissolved in Laemili buffer containing 0.1 mM DTT and run in a one-dimensional SDS gel electrophoresis gel at 200 V for 50 min. The gel was fixed with methanol and stained with Coomassie for protein visualization. Each gel lane was sliced into 30 segments, and each slice was digested with trypsin. After washing and dehydration with acetonitrile, and rehydration with ammonium bicarbonate, extracted peptides were then completely dried in a SpeedVac (ThermoScientific, Waltham, MA).

### Mass Spectrometry and protein identification

MS was carried out as previously described[14]. Briefly, dried peptides were resuspended in trifluoroacetic acid (TFA) and 6μL was injected and loaded onto a C18 trap column. The sample was subsequently separated by a C18 reverse-phase column. The mobile phases consisted of water with 0.1% formic acid (A) and 90% acetonitrile with 0.1% formic acid (B). A 65-min linear gradient from 5 to 60% B was used. Eluted peptides were introduced into the mass spectrometer via a 10-μm silica tip (New Objective Inc., Ringoes, NJ) adapted to a nano-electrospray source (Thermo Fisher Scientific). The spray voltage was set at 1.2 kV and the heated capillary at 200°C. The linear trap quadrupole (LTQ) mass spectrometer (ThermoFisher Scientific) was operated in data-dependent mode with dynamic exclusion in which one cycle of experiments consisted of a full-MS (300–2000 m/z) survey scan and five subsequent MS/MS scans of the most intense peaks. Pathways enriched with the proteins were generated by Ingenuity Pathways Analysis (IPA, version 8.5, Ingenuity Systems, Redwood City, CA).

### Cytokine Multiplex assay

Because proteins less than 20kDa in size are typically too small to be detected by one-dimensional gel mass spectrometry proteomics approaches, we also analyzed MEE samples (n = 49) for quantitative levels of IL10, macrophage derived chemokine (MDC), IL13, IL17A, IL1β, IL6, IL8, RANTES, TNFα, and VEGF using a commercially available multiplex magnetic bead immunoassay (Millipore, Billerica, MA, USA) according to the manufacturers’ instructions using provided standards and quality controls as previously described[27]. For this assay, MEE aliquots were further diluted in 5 ml of PBS.

### Immunofluoresence

Freshly obtained MEE samples were gently spread directly over a glass slide and allowed to dry for 2 to 4 hours on ice, before fixation in paraformaldehyde 3% for 20 min and storage at 4°C in PBS 1X until immunostaining was performed. Cells were permeabilized with PBS/Tween 20 0.05% for 5 min; non-specific sites were saturated using 0.01% PBS/Tween-20/3% Bovine Serum Albumin for 30 min. This solution was also used to incubate primary anti-human antibodies for -neutrophil elastase (ab68672, Abcam, Cambridge, MA), Citrullinated H3 (ab5103, Abcam, Cambridge, MA), MUC5B (sc-135508, Santa Cruz, Dallas, TX), Lysozyme (EC3.2.1.17, Dako, Carpinteria, CA), Lactoferrin (ab77780, Abcam, Cambridge, MA), Myeloperoxidase (ab25989, Abcam, Cambridge, MA), S100A8/9 (ab17050, Abcam, Cambridge, MA), S100A9 (sc-53187, Santa Cruz, Dallas, TX), SPLUNC (kindly provided by Dr Colin Bingle, The University of Sheffield Medical School) at 1/500 dilution and the secondary antibodies anti-goat Alexa 488 A11055 oranti-rabbit Alexa A10042 at 1/500 (Invitrogen, Carlsbad, CA). Three washes of 30 min were done in order to reduce the non-specific signal of the antibodies. 4',6-diamidino-2-phenylindole(DAPI) at 1:8000 in PBS was finally used to stain DNA. The chambers were removed and the slide was mounted using Permount mounting medium (Fisher Scientific, Suwanee, CA) with a coverslip. Slides were stored at 4°C in dark before immunofluorescent analysis using an Olympus FV1000 confocal microscope (Olympus, Rocklin, CA).

### Western Blot Analyses

A previously described protocol was used for the western blotting[28] of non-mucin proteins. Briefly, 15μl of each effusion sample was separated by electrophoresis on NuPAGE Novex 4–12% Bis-Tris gels (Life technologies, Carlsbad, CA). The molecular weight marker Kaleidoscope was used as a standard (Bio-Rad, Hercules, CA). The proteins were then transferred to a nitrocellulose membrane (Invitrogen). Membranes were blocked with 5% non-fat dry milk in PBS with 0.05% Tween-20 (PBST), and incubated with the same primary antibodies at the same concentration as described for IF. Secondary antibody anti-rabbit at 1:5000 coupled to horseradish peroxidase (SigmaAldrich, St. Louis, MO). Detection was performed with a SuperSignal^®^ West Dura Extended Duration Substrate kit (Pierce, Rockland, IL) according to the manufacturer’s instructions.

For mucins, western blot analysis was performed as previously described[14]. Briefly, samples containing 50 μg total proteins were separated by electrophoresis on a 1.0% agarose (molecular biology grade, Life technologies, Carlsbad, CA) gels. 1X Tris-acetate-EDTA (TAE) containing 0.1% SDS, 1mM EDTA, and 40mM Tris acetate (pH 8.0) was used as the electrophoresis buffer. Samples were solubilized in sample buffer, denatured at 95°C for 10 minutes and loaded into a horizontal gel apparatus. Electrophoresis was performed at 35 V for 2 hrs, and at 15 V overnight. Proteins were transferred under positive pressure onto a polyvinylidine difluoride (PVDF) membrane (Millipore, Bedford, MA). After drying the membrane and activating in Methanol, it was incubated for 1 hr at RT in 5% non-fat dry milk in PBS with 0.05% Tween-20 (PBST) and then in a 2.5% milk solution with a rabbit polyclonal anti-MUC5B antibody H-30 (Santa Cruz, CA) at 1:300 dilution. The secondary antibody goat-anti rabbit coupled to HRP at a 1:20,000 dilution (KPL, Inc., Gaithersburg, MD) was used for the immunodetection. Chemiluminescence was carried out using SuperSignal^®^ West Dura Extended Duration Substrate kit (Pierce, Rockland, IL) according to the manufacturer’s instructions.

### Neutrophil elastase enzymatic activity assay

Neutrophil elastase protein activity was assayed with the Elastase Substrate I, Colorimetric (Millipore, Bedford, MA). The substrate was reconstituted in 0.1M Hepes, 0.5M NaCl and 10% dimethylsulfoxide at 1 mg/ml as performed previously[29]. 200μl of this substrate solution was added to each well of a transparent 96 well plate (Greiner Bio-One, Monroe, NC) and 5 to 10μl of MEEs, neutrophil lysate or PBS was added to the wells. After 6 hours of incubation at 37°C, the optical density (O.D.) was read at 410nm with an xMark microtiter plate reader (Biorad, Richmond, CA).

### DNA Quantification

DNA from 200 uL of middle ear effusions was purified using the QiaAmp mini kit (Qiagen, Valencia, CA) prior to electrophoresis in 1% Agarose Gels run at 100 V in 1X TAE buffer containing ethidium bromide for visualization under ultraviolet lighting. Quantification of relative DNA concentrations in each effusion sample was performed by the Qubit double stranded DNA kit (Invitrogen, Carlsbad, CA).

### Next Generation Sequencing (NGS)

Quality control was performed on 49 DNA samples from middle ear effusion using the Qubit 2.0 fluorometer system (Life Technologies, Boston, MA) and the nanodrop. These samples were further processed for shotgun sequencing using a Truseq Nano prep kit (Illumina, San Diego, CA). Genomic libraries were validated (concentration and size) with the Qubit and 2100 Bioanalyzer. Forty-four samples passed the validation and were sequenced on the Nextseq 500 using the 2x151 basepair paired end protocol, after an initial loading titration trial sequencing run. Raw reads (FASTQ format) were preprocessed (QC) using PRINSEQ-lite 0.20.4 (trimming reads and bases < 25 PHRED, minimum length = 100, removing exact duplicates, reads with undetermined bases, and low complexity reads using Dust filter = 25)[30]. Filtered (paired and unpaired reads) were aligned to the human genome (hg19) using Bowtie2 (—very-sensitive)[31].

## Results

### MEEs are characterized by a mucosal innate immunity response, with abundant neutrophil markers

The entire middle ear proteome from 6 middle ear effusions was previously reported by out group[14]. This protein list was validated in a set of 3 new middle ear effusions, which revealed 429 unique proteins (S1 Table). The 30 most abundant proteins found in the COM fluid samples (n = 9) (excluding immunoglobulins, blood proteins and keratin contaminants) by average MS/MS spectral counts are listed in Table 1. To demonstrate the specificity of these proteins to MEE, rather than to serum contaminant, the average peptide counts (PC) of these proteins in serum are also listed. Mucosal innate immunity proteins with the highest PC included: Long palate, lung and nasal epithelium carcinoma-associated protein 1 (LPLUNC), Lactotransferrin (LTF), mucin 5B (MUC5B) and S100 proteins A9 and A8. These proteins were nearly universally absent in serum controls.

**Table 1:** 30 most abundant proteins detected by MS/MS*.

Uniprot ID	Protein name	serum	average MEEs
Q8TDL5	Long palate, lung and nasal epithelium carcinoma-associated protein 1 (LPLUNC1)	0	284
P02788	Lactotransferrin (LTF)	0	211
Q9HC84	Mucin-5B (MUC5B)	0	174
P62736	Actin, aortic smooth muscle (ACTA2)	1	118
P06702	Protein S100-A9 (S100A9)	5	174
P05109	Protein S100-A8 (S100A8)	3	129
P62805	Histone H4 (HIST1H4A)	1	110
P61626	Lysozyme C (LYZ)	9	63
P60709	Actin, cytoplasmic 1 (ACTB)	0	62
P02679	Fibrinogen gamma chain (FGG)	0	61
Q9NP55	Protein Plunc (PLUNC)	0	60
P05164	Myeloperoxidase (MPO)	0	53
P02675	Fibrinogen beta chain (FGB)	0	41
P08311	Cathepsin G (CTSG)	0	28
Q96QV6	Histone H2A type 1-A (HIST1H2AA)	0	28
P04406	Glyceraldehyde-3-phosphate dehydrogenase (GAPDH)	0	27
P08246	Leukocyte elastase (ELA2)	0	27
P01833	Polymeric immunoglobulin receptor (PIGR)	0	26
Q9UGM3	Deleted in malignant brain tumors 1 protein (DMBT1)	0	25
P06733	Alpha-enolase (ENO1)	0	23
P33778	Histone H2B type 1-B (HIST1H2BB)	0	22
Q562R1	Beta-actin-like protein 2 (ACTBL2)	0	21
P80188	Neutrophil gelatinase-associated lipocalin (LCN2)	0	21
P12814	Alpha-actinin-1 (ACTN1)	0	21
P04083	Annexin A1 (ANXA1)	0	20
P08670	Vimentin (VIM)	0	19
P30740	Leukocyte elastase inhibitor (SERPINB1)	0	17
Q9BYX7	Beta-actin-like protein 3 (ACTBL3)	0	17
P02671	Fibrinogen alpha chain (FGA)	5	22
P02790	Hemopexin (HPX)	9	16

* This table shows the peptide counts of the serum sample and the average peptide count of 9 middle ear effusions (MEEs) for each protein.

Due to their very high abundance in COM samples, immunoglobulins as well as complement proteins are summarized in Table 2. 12 complement proteins were detected in the MEE (PC average higher than 2) and 16 were detected in serum. All complement proteins were more abundant in serum than in MEE samples, with a PC percent of 3.8% for MEE and 8.1% for serum. On the other hand, immunoglobulins were more abundant in the MEE samples, representing 8.6% of the total PC whereas the serum sample was 3.0%. Among the heavy chains, IgGs were the most abundant for the MEE and serum. IgA, as expected, was markedly more abundant in the MEEs. IgM, unknown Ig heavy chains and IgJ were also detected. A very low abundance of IgD was detected, and no IgE was found.

**Table 2:** Complement and immunoglogulin (Ig) proteins detected by MS/MS.

**Complement proteins**			
**Uniprot** ID	**Protein name**	**serum**	**Average MEEs**
P01024	Complement C3 (C3)	621	162
P0C0L4	Complement C4-A (C4A)	181	28
P00751	Complement factor B (CFB)	78	17
P08603	Complement factor H (CFH)	61	15
P01031	Complement C5 (C5)	22	0
P13671	Complement component C6 (C6)	14	1
P06681	Complement C2 (C2)	13	1
P09871	Complement C1s subcomponent (C1S)	10	0
P10643	Complement component C7 (C7)	9	2
P00736	Complement C1r subcomponent (C1R)	7	0
P07360	Complement component C8 gamma chain (C8G)	6	3
P05156	Complement factor I (CFI)	6	2
P02746	Complement C1q subcomponent subunit B (C1QB)	5	3
P02747	Complement C1q subcomponent subunit C (C1QC)	4	0
P02748	Complement component C9 (C9)	2	1
P0C0L5	Complement C4-B (C4B)	2	0
Q03591	Complement factor H-related protein 1 (CFHR1)	1	0
P07358	Complement component C8 beta chain (C8B)	0	0
P00746	Complement factor D (CFD)	0	0
P07357	Complement component C8 alpha chain (C8A)	0	0
	% PC	8.1	3.8
**Ig light chains**			
		**serum**	**Average MEEs**
	Ig kappa	358	501
	Ig lambda	23	33
	% PC	3.0	8.6
**Ig heavy chains**			
		**serum**	**Average MEEs**
	IgG	573	867
	IgA	106	266
	IgM	58	72
	Ig heavy chain	33	77
	IgJ	2	19
	IgD	4	3
	% PC	6.0	20.9
	Total % PC for Ig	9.0	29.4

Surprisingly, five actin and three histones were detected in the high abundance list, but only in the MEE samples and not in serum. Additionally, the three neutrophil/leukocyte proteins in the highly abundant list were present only in MEE and not in serum.

### Identification of NET markers in Proteomic Data from MEE

We hypothesized the presence of NETs as an explanation for the high abundance of actin, histones and neutrophil markers in MEE. Table 3 shows a list of proteins from our proteomic analysis that were previously reported to characterize NETs [32] and shows that these markers, including antibacterial proteins like lactotransferrin (LTF), lysozyme C (LYZ), myeloperoxidase (MPO) and leukocyte elastase (ELANE or NE), account for 17% of all mapped PC of the MEE samples whereas they were consistently absent in serum. A more detailed list of the histone proteins shows a predominance of histone H4, a lower PC for H2 proteins, and minimal PC for H1 and H3. Overall, histone proteins accounted for 2.8% of the PC in MEEs and 0% in serum.

**Table 3:** Proteins implicated in NETs detected by MS/MS.

NET proteins			
Uniprot ID	Protein name	serum	Average MEEs
P02788	Lactotransferrin (LTF)	0	211
P06702	Protein S100-A9 (S100A9)	5	174
P05109	Protein S100-A8 (S100A8)	3	129
P62805	Histone H4 (HIST1H4A)	1	110
P61626	Lysozyme C (LYZ)	9	63
P60709	Actin, cytoplasmic 1 (ACTB)	0	62
P05164	Myeloperoxidase (MPO)	0	53
P08311	Cathepsin G (CTSG)	0	28
P04406	Glyceraldehyde-3-phosphate dehydrogenase (GAPDH)	0	27
P08246	Leukocyte elastase (ELA2)	0	27
P06733	Alpha-enolase (ENO1)	0	23
P33778	Histone H2B type 1-B (HIST1H2BB)	0	22
P80188	Neutrophil gelatinase-associated lipocalin (LCN2)	0	21
P12814	Alpha-actinin-1 (ACTN1)	0	21
P30740	Leukocyte elastase inhibitor (SERPINB1)	0	17
P13796	Plastin-2 (LCP1)	0	14
P07737	Profilin-1 (PFN1)	0	13
P35579	Myosin-9 (MYH9)	0	12
P04075	Fructose-bisphosphate aldolase A (ALDOA)	0	5
P26038	Moesin (MSN)	0	5
P06744	Glucose-6-phosphate isomerase (GPI)	0	5
P20160	Azurocidin (AZU1)	0	4
P37837	Transaldolase (TALDO1)	0	4
P17213	Bactericidal permeability-increasing protein (BPI)	0	4
P29401	Transketolase (TKT)	0	4
P04908	Histone H2A type 1-B/E (HIST1H2AB)	0	4
P62807	Histone H2B type 1-C/E/F/G/I (HIST1H2BC)	0	3
Q96A08	Histone H2B type 1-A (HIST1H2BA)	0	2
	% PC		17.0

### Characterization of NETs in MEE

**Fig 1:**
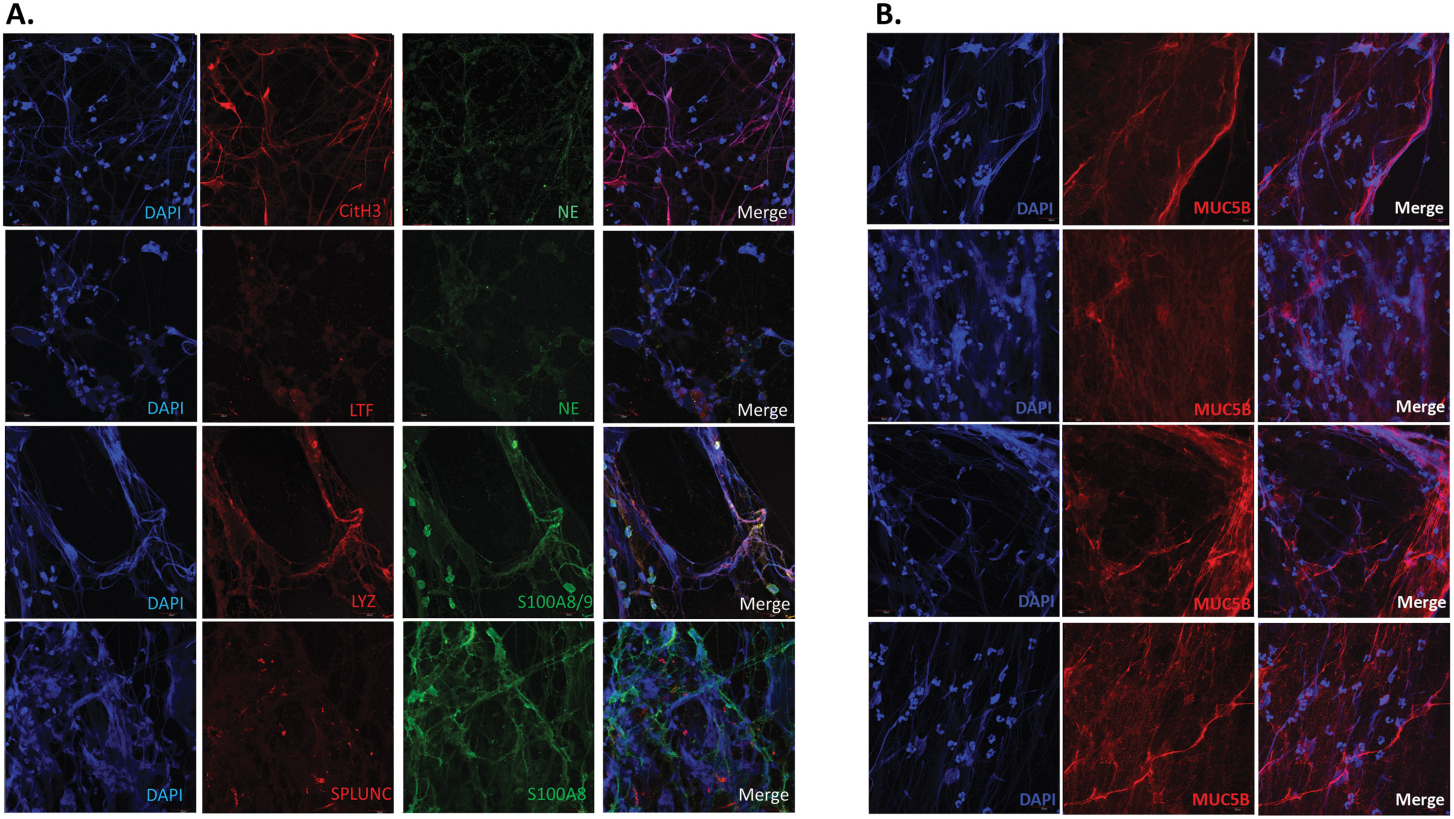
Immunofluorescence of MEE. A. Visualization of innate immunity and neutrophil markers by confocal microscopy after immunolabelling. Citrullinated H3 (CitH3), Neutrophil elastase (NE), lysozyme (LYZ), S100A8, S100A9, lactotransferrin (LTF), short Palate Lung And Nasal Epithelium Clone Protein (SPLUNC) were labeled using specific antibodies. The DNA was stained using DAPI. Mounted slides were then observed with a confocal microscope. Each row represnets a separate middle ear effusion sample. B. Visualization of NET extracellular DNA association with mucin MUC5B. The DNA was stained using DAPI. MUC5B was found to associate with extravasated, but not nuclear DNA. The top two rows are from one MEE, while the bottom two are from a separate MEE sample.

Given the above mentioned results and findings we postulated that visualization of fresh, independent MEE samples would reveal a copious amount of NETs. Through DAPI staining of DNA and antibody staining of specific proteins, IF unequivocally revealed abundant extravasation of DNA from cellular structures resulting in variable stranding of DNA in MEE specimens. Validation with NE, LYZ, and LTF confirmed these cells to be neutrophils. Colocalization of extracellular DNA strands with citrullinated histone-3 (CitH3) confirmed these networks of extravasated DNA and extracellular proteins in MEE fluid are indeed NETs, as the citrullination of histones is characteristic of NET DNA release[23,33] (Fig 1A) Notably, other innate immune mediators, such as Protein S100 and PLUNCs were also found to be associated with the NETs. Importantly, mucin MUC5B was found to associate with the NETs, ostensibly forming a thick mesh of extracellular DNA, and mucous strands (Fig 1B). Notably, mucin MUC5AC staining with IF was uniformly absent in these specimens (data not shown). Table 4 summarizes the IF findings in 9 MEE samples (unique samples,e.g. not used for proteomics). In summary, all tested samples showed evidence of NET formation, comprised by extracellular DNA, lysozyme, neutrophil elastase, myeloperoxidase, citrullinated H3 (Cit H3), and lactotransferrin.

**Table 4:** Summary of IF results.

	DNA filaments	Neutrophil elastase	Citrulinate H3	MPO	Lactotransferrin	Lysozyme	S100A8/9	MUC5B	MUC5AC	LPLUNC
‘n’ samples postive/tested	9/9	6/6	5/5	1/1	1/1	3/3	6/6	8/8	5/8	1/1
% positive	100	100	100	100	100	100	100	100	63	100

**Fig 2:**
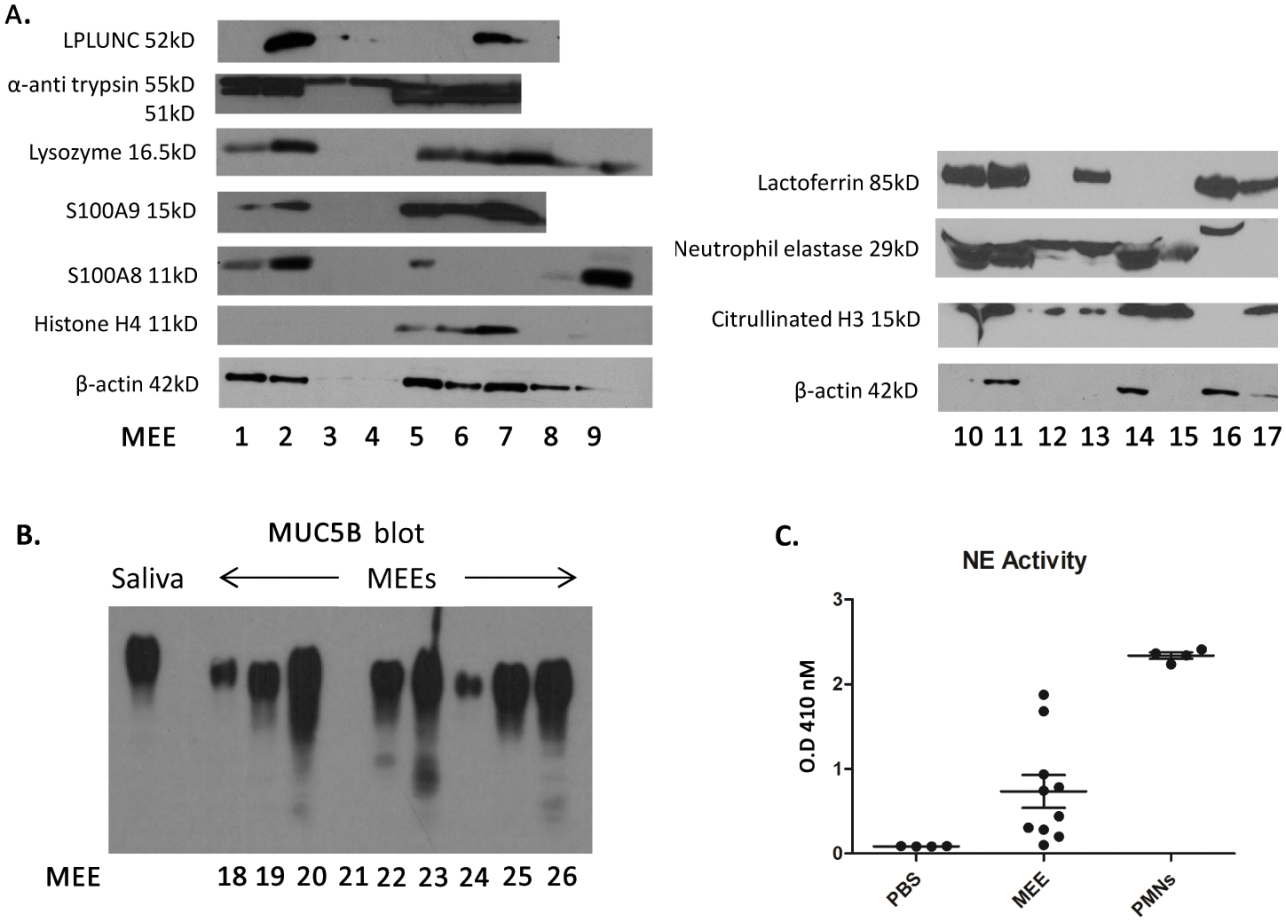
Western Blot analysis of MEE. **A.** 10μl of middle ear effusions (MEEs) were loaded in a polyacrylamide gel before electrophoresis. Proteins were transferred on a nitrocellulose membrane and exposed to specific antibodies to reveal proteins of interest (as listed in the figure axis). A consistent presence of typical neutrophil markers was noted across tested samples, validating the proteomic results. Of note, not all mediators were identified in each effusion presumably due to protein degradation. **B.** 10μl of middle ear effusions (MEEs) were loaded in an agarose gel and electrophoresed before transfer to a PVDF membrane and exposed to aMUC5B antibody, revealing a uniform presence of this mucin glycoprotien across tested MEE samples (as expected). **C.** 10μl of MEEs, neutrophil lysates (PMNs) or PBS were incubated in Substrate Elastase I for 6 hours. The optical density (O.D.) was then read at 410nm to determine the apparition of the resultant chromophore. This demonstrates increased neutrophil elastase (NE) activity in MEE over background diluant levels.

Immunoblotting was subsequently used to further evaluate the presence or absence of NET markers in independent samples. Western blot analyses demonstrated the presence of NET markers lactotransferrin, CitH3, Histone H4, lysozyme, and neutrophil elastase, as well as the innate immune mediators LPLUNC, α-antitrypsin, and proteins S100A9 and A8 in a majority of the samples evaluated. (Fig 2A). As expected, mucin MUC5B, was also abundant in an independent set of MEE samples (Fig 2B). Moreover, neutrophil elastase activity assays of independent MEE samples further validated the presence of neutrophil elastase (Fig 2C). This activity was shown to be higher than the negative control PBS but lower than the positive control (lysate of neutrophils).

### MEEs show a high content of human origin large DNA fragments

**Fig 3:**
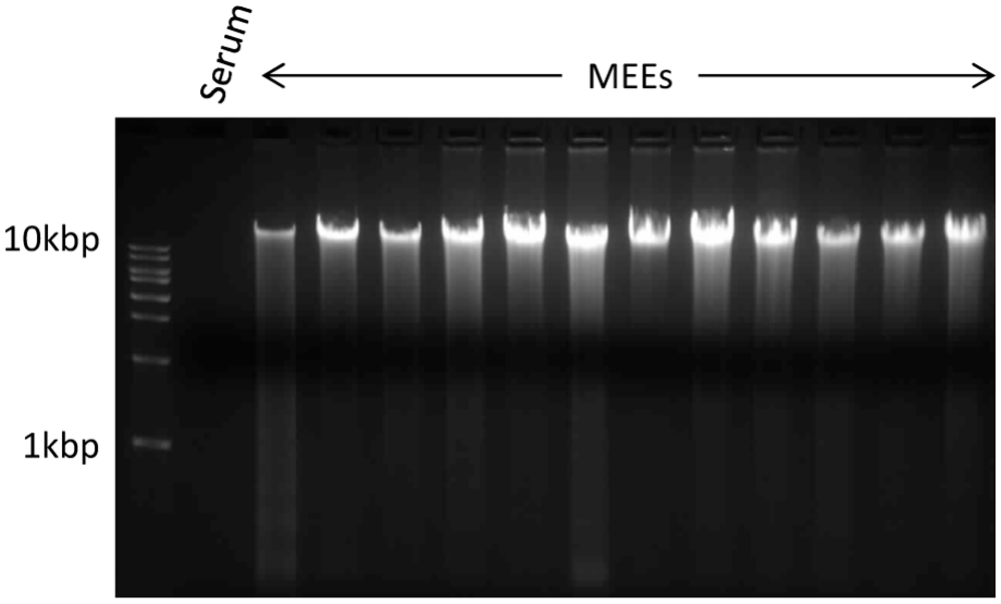
Middle ear effusions (MEEs) DNA gel electrophoresis. DNA of twelve MEEs and one serum sample (considered as a negative control) were extracted with the QiaAmp DNA mini kit. The DNA concentration was measured with the Qubit double stranded DNA kit, and 1μg of each sample (except the serum because of its very low DNA concentration) was loaded in each well as well as 10μl of DNA ladder in a 1% agarose gel containing ethidium bromide. After 2hours of electrophoresis at 100V the gel was observed in a UV box.

In order to determine whether the DNA present in MEE from COME patients (Fig 1) was more consistent with NETosis DNA or apoptosis/necrosis DNA, we isolated DNA and performed agarose gel electrophoresis to visualize DNA sizes (Fig 3). The DNA purified from the same volume of MEEs varied between 49.6 ng/μl and 324 ng/μl, showing a variability in DNA content depending on the patient. Comparatively the DNA concentration from the serum control sample was 5 ug/ml. Importantly, DNA isolated from MEE was found to be predominantly large band slightly higher than the 10kbp DNA ladder marker. Additionally, in some samples, the DNA did not enter the gel. Most of the DNA was not laddered or fragmented, suggesting that apoptosis and necrosis are not the primary source of the DNA. Importantly, NGS with Kraken software[34] for analysis of DNA genome source revealed an average of 12.2 million reads (paired and unpaired) per sample aligned to the human genome, while an average of 1.1 million reads were filtered off (non-human reads) (Table 5). As such, one can conclude that 91.3% of the DNA extracted from the MEE were from human cell origin. The whole genome sequecing data for all samples has been uploaded on to the National Center for Biotechnology Information (NCBI) website under project ID SRP069302.

**Table 5:** Summary of DNA origin in MEE by next generation sequencing.

	Mean reads	Mean host reads	Mean % host reads	Mean non host reads	Mean % non host reads
n = 42	13,287,946	12,181,318	91.28%	1,106,628	8.72%
SD	4,391,051	4,187,744	2.88%	394,380	2.88%

### Pathway analysis of the MEE proteomic dataset

**Fig 4:**
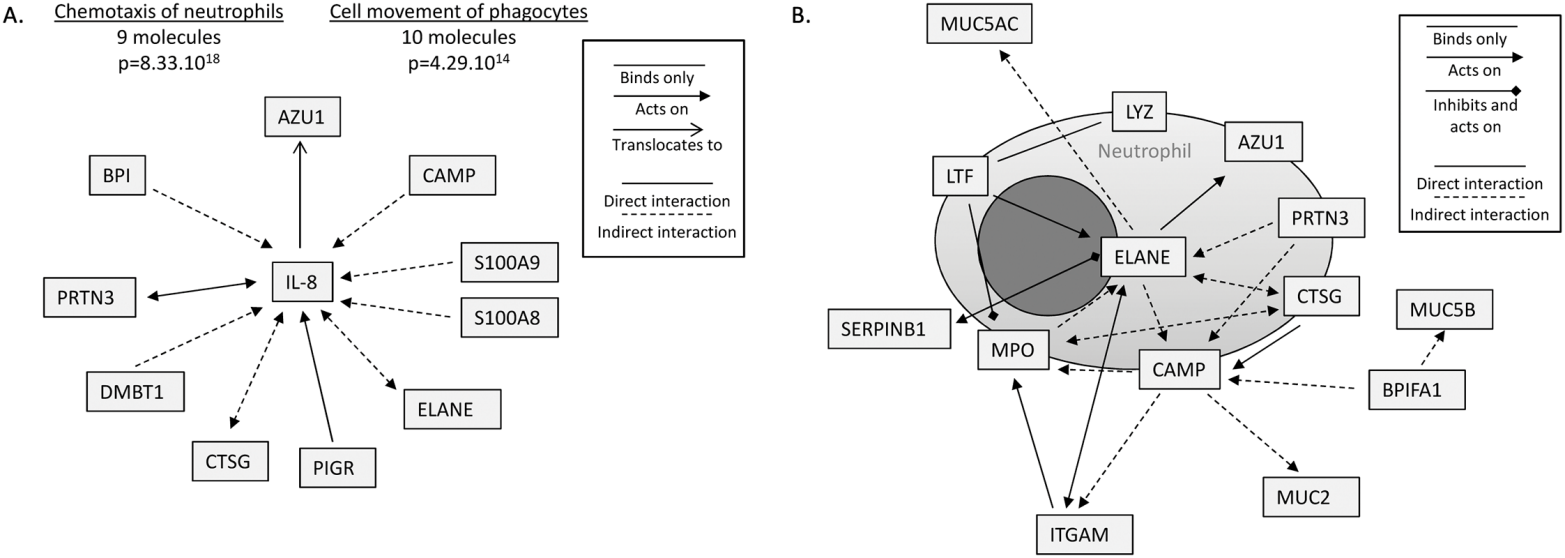
Relationships between proteins detected in the middle ear effusions (MEEs) and functions associated, determined by Ingenuity Pathway Analysis (IPA). **A.** 10 most abundant proteins realted to the pro-inflammatory cytokine IL-8 and associated molecular and cellular functions. The relationships between IL-8 and the proteins detected in MEE samples are represented by lines and arrows as detailed in the legend. The functions assigned by IPA along with the number of molecules implicated and the p value are listed on top. (LYZ lysozyme; AZU1 azurocidin; PRTN3 proteinase 3; CTSG Cathepsin G; CAMP cAMP receptor protein; MPO myeloperoxidase; LTF lactoferrin; MUC5AC mucin 5AC; MUC5B mucin 5B; MUC2B mucin 2B; ELANE neutrophil elastase; BPIFA1 BPI Fold Containing Family A, Member 1; ITGAM integrin alpha M; SERPINB1 leukocyte elastase inhibitor; S100A8/9 S100 calcium-binding protein A8/9; PIGR Polymeric immunoglobulin receptor; DMBT1 Deleted in Malignant Brain Tumors-1. **B.** Neutrophil proteins detected in the MEEs centered/focused on ELANE. Relationships between these and other non-neutrophilic proteins are represented by lines an arrows as detailed the legend.

Ingenuity Pathway Analysis (IPA) (QIAGEN, Redwood City, CA) was then used to analyze the relationships between the proteins of the MEE proteomic dataset. Notably, abundant MEE proteins were predicted to be directly linked to the action of IL-8, which given these relationships, was determined to most likely be involved in MEE for the process of neutrophil chemotaxis as well as for the movement of phagocytes/myeloid cells (p = 8.33x10^-18^ and p = 4.29x10^-14^ respectively) (Fig 4A). Almost all the identified mediators directly or indirectly act on IL-8, and are proteins produced by neutrophils such as ELANE (neutrophil elastase), CTSG (cathepsin G), AZU1 (azurocidin) and PRTN3 (proteinase 3); or proteins implicated in the mucosal innate immune response as S100A8 and A9, PIGR (polymeric immunoglobulin receptor) and BPI (bactericidal permeability increasing proteins). These results highly suggest that proteins in the secretions attract neutrophils to the middle ear epithelium that in turn sustain the production of IL-8 and inflammation. Fig 4B, which is centered on NE, also shows that neutrophil proteins have direct or indirect relationships with each other and indirectly act on the mucins MUC5B, MUC5AC and MUC2, suggesting a predicted interaction between NETs and the mucoid status of MEEs in COM.

### MEEs contain high levels of inflammatory mediators, particularly IL-8

**Fig 5:**
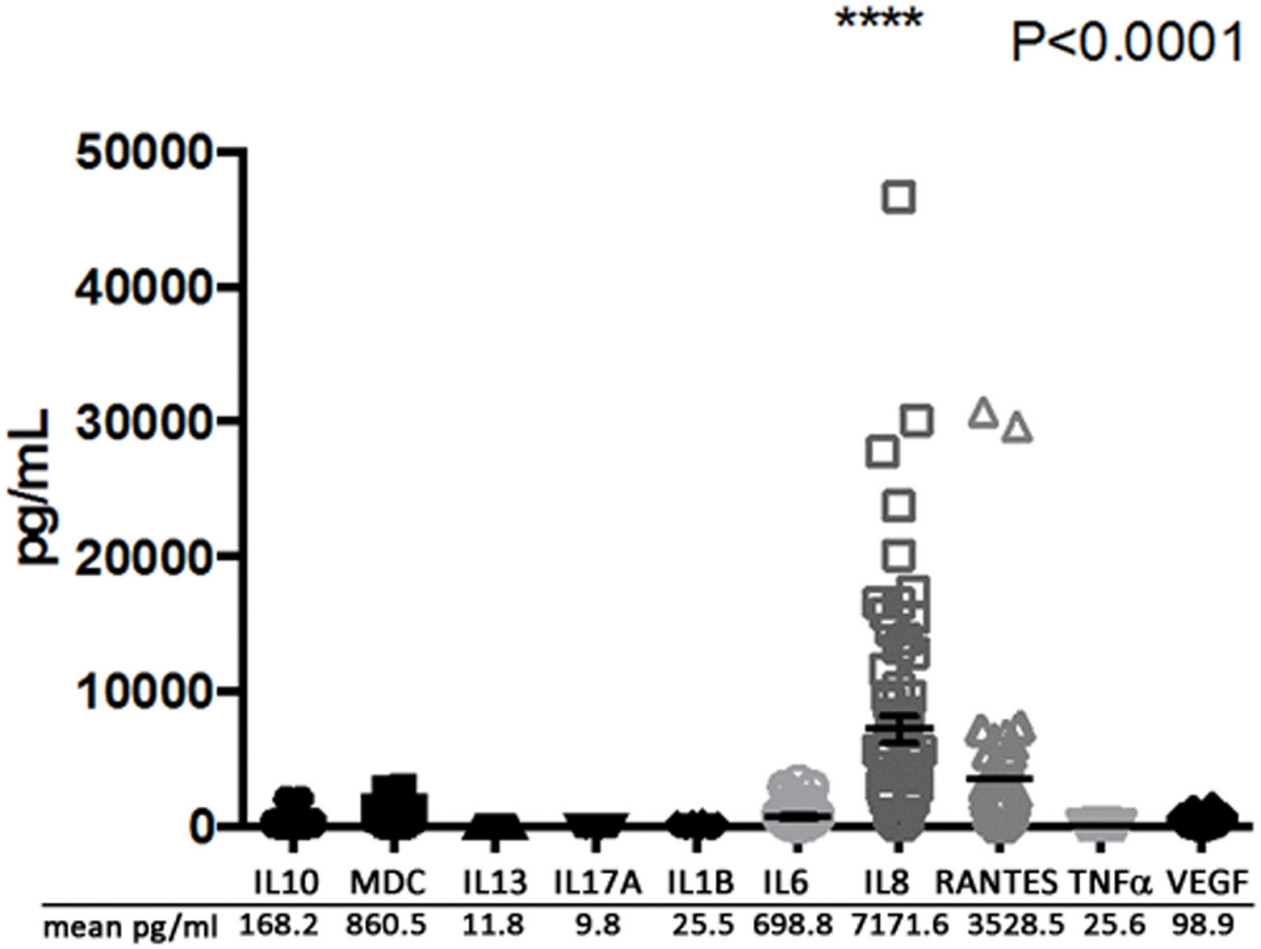
Inflammatory mediator concentrations in 49 middle ear effusions (MEEs) from COM patients. A Luminex multiplex assay was performed with 50 different MEEs to determine the concentration of 10 inflammatory mediators: IL1B/6/8/10/13/17A interleukin 1B/6/8/10/13/17A; MDC macrophage derived cytokine; RANTES regulated on activation, normal T cell expressed and secreted; TNFα tumor necrosis factor alpha; VEGF vascular endothelial growth factor. The individual results are represented as shapes of each mediator and the mean concentration is listed below the graph. IL8 concentration was statistically higher by ANOVA (p<0.0001) than the concentration of the other mediators.

Because proteomic techniques are limited in their ability to detect small molecules such as cytokines, we performed a multiplex assay to quantify the levels of a group of representative Th1, Th2 and other cytokines that have previously been associated with COM[35,36] (Fig 5). As predicted by IPA, IL8 was found to have the highest abundance of all measured cytokines with an average concentration of 7172 pg/ml. This finding is in line with the IPA analysis of the COM proteome, linking the MEEs protein dataset with this pro-inflammatory cytokine and its ability to chemoattract and activate neutrophils. The IL-8 concentration was shown to be nearly 100 fold higher than all other cytokines (p<0.0001) except for RANTES, which showed an average level of 3528 pg/ml. Other mediators such as MDC and IL-6 were in high concentrations in the effusions (860.5 pg/ml and 698.8 pg/ml respectively), whereas IL10, IL13, IL17A, IL1β, TNFα and VEGF were under 200 pg/ml.

## Discussion

This study comprehensively validates the fact that NETs are present in the MEE of pediatric patients and reports for that proteins derived from NETS are the predominant innate immune response mediators in COM, providing a detailed proteomic analysis of COM MEEs.

Analysis of the MEEs proteome revealed a majority of proteins implicated in mucosal innate immune responses as observed in proteomic analysis of airway secretions *ex vivo and* airway epithelial cell secretions *in vitro*. Joo et al. compared their proteomic analysis of normal human airway gland secretions with proteomic studies of of bronchial alveolar lavage fluid, apical fluid of primary human tracheobronchial epithelial cell cultures, and nasal lavage fluid and demonstrated an overall similarity in the dataset profiles from each of the sample sources[37]. Our MEEs samples also show an abundance of the same protective proteins of innate mucosal immunity. Among them, mucins and especially MUC5B, PLUNC proteins, S100A8/A9, histones, complement proteins, DMBT1, neutrophil gelatinase associated lipocalin (LCN2), annexins, extracellular matrix proteins such as fibrinogen and laminins, polymeric immunoglobulin receptor and lactotransferrin are also common to secretion samples from bronchial epithelium, sinuses, and middle ear sources, highlighting a uniform innate immune response in the respiratory tract epithelium[14,38,39,40]. It is interesting to note that airway epithelia already secrete these proteins even in the absence of pathogens whereas the middle ear develops this response over time during/after infection. This is supported by the fact that mucoid samples are mostly observed at chronic stages of OM whereas acute stages show mostly serous or purulent middle ear fluid[41] and that COM is characterized by a remodeling of the middle ear epithelium into a pseudostratified mucociliary epithelium similar to the bronchial epithelium, able to secrete mucins[16,17,42].

Our MEE dataset was further characterized by the abundance of proteins produced by neutrophils, such as neutrophil elastase (NE, or leukocyte elastase ELANE), myeloperoxidase (MPO), Cathepsin G (CTSG), Lactotransferrin (LTF) but also intracellular proteins typically not expected in such abundance in secretions: actin and histone H4. This finding led us to investigate the presence of NETs in the MEE of patients with COME, as recently reported [21,22]. In 2004, NETs were first described and reported to represent a fundamental innate immune process focused on eliminating bacteria from mucosal membranes[23]. During NETosis, the neutrophil nuclear lobules fuse, partially decompacting DNA due to citrullination of histones. This allows for the DNA to bind antimicrobial proteins such as neutrophil elastase (NE), myeloperoxidase (MPO), lysozyme (LYZ) among others[32,33]. Neutrophils then release this DNA into the extracellular space whereby it reaches pathogens and mediates direct bacterial killing and facilitates phagocytosis. NETs function by binding microorganisms, preventing them from spreading, and ensuring a high local concentration of antimicrobial agents to degrade virulence factors and kill bacteria. Neutrophil function and NETs are known to be critical components of immune defense. For example, patients with chronic granulomatous disease have impaired neutrophil function, cannot form NETs, and as such are tremendously susceptible to lethal infections. An excess of NETs, however, has been reported to contribute to the pathology of a number of diseases. For example, NETs have been shown to exert damaging inflammation and tissue injury including inducing epithelial and endothelial apoptosis[43]. Furthermore, NETs can incorporate into bacterial biofilms, and in some cases effectively allow for bacteria to ‘hide’ from the immune system or from bactericidal antibiotics[44]. In fact, in chinchilla models of OM, NETs have been shown to not be effective at clearing NTHi and their presence actually positively strongly correlates with bacterial load in the middle ear[44].

Of particular note, IL-8 was found to be by far the most abundant cytokine detected by multiplex assay in out MEE samples. This is of major relevance to the finding of NETosis in MEE, because not only is IL-8 a well known chemoattractant for neutrophils, its presence has been shown to positively correlate with the abundance of NETs in bronchial fluids[45]. Further, IL-8 has been shown to induce *in vitro* NETosis in neutrophils from healthy subjects [46]. Aged mice skin cells, show a decreased ability to secrete Cxcl2 upon methacillin resistant *S*. *Aureus* (MRSA) challenge, (a murin IL8 functional homologue) with a parallel reduced ability for neutrophils from aged mice to form NETs kill MRSA[47]. Finally, in a mouse model of abdominal sepsis, neutrophil derived NETs have the ability to induce Cxcl2 formation from macrophages[48], suggestting that the formation of NETs may be a pro-inflammatory process in and of itself. Given this published data, considering the abundance of NET proteins in our proteomics dataset, it is not surprising that IPA revealed that a majority of abundant MEE proteins are directly linked to the action of IL-8.

It thus appears that rapid clearance of NETs from mucosal surfaces the airways is critical in preventing their deleterious effects. In the lung, the effective clearance of NET structures is a critical process in the maintenance of healthy airways. The lack of appropriate NET homeostasis, with accumulation of bacteria, extracellular DNA and NET-associated enzymes such as MPO and elastases, worsening of lung inflammation and tissue damage has been demonstrated in diseases such as CF[49] and asthma[50]. In COPD, a higher amount of NETs in the airways has been demonstrated to worsen airflow and to directly correlate with disease severity[51]. In terms of the middle ear, inadequate Eustachian tube function (as is common in young infants and toddlers) is quite likely to contribute to lack of NET clearance. Furthermore, accumulation of large macromolecular mucin glycoproteins potentiate difficulties with NET accumulation in the middle ear cleft. Indeed, our data demonstrates an association of NET matrices with mucin MUC5B. How mucins contribute to the process of NETosis in terms of either bacterial kill, or NET accumulation has not been studied. Treatment strategies which aim to modulate NET accumulation are increasingly gaining traction as useful therapeutic approaches for chronic respiratory diseases and have even been proposed in recurrent acute OM[22].

Our work confirms and establishes the presence of abundant NETs in COM fluids using immunofluorescence analysis of fresh MEEs. This is important in order to avoid sample degradation and spontaneous neutrophil activation due to temperature among other factors. All the mucoid MEEs were characterized by the presence of cells releasing high amounts of DNA in the extracellular space, which co-localized with CitH3 and other antibacterial proteins as lysozyme, lactotransferrin, neutrophil elastase which are proteins known to be characteristic of NETs[52] along with other innate immune proteins as MUC5B, S100A8, S100A9 and SPLUNC. Calprotectin, a dimer of S100A8 and S100A9, has also been shown to bind to NETs and is reportedly required for NET antifungal activity[52], likely making it important in OM. SPLUNC, another abundant protein in the MEEs has been demonstrated to contribute to pulmonary host defense, and its depletion in mice leads to middle ear infection[53,54]. Finally, MUC5B, the predominant mucin in human COM fluid[14] has also shown to be required for airway defense, with its absence leading to severe middle ear infection in mice[55]. The findings build on previous work published about NETs in OM by validating NETs in MEE through a larger number of typical NET markers (such as the above mentioned CitH3 and myeloperoxidase) hitherto not studied in OM, but nonetheless of critical importance in the definitive identification of NET structures[43,56].

**Fig 6:**
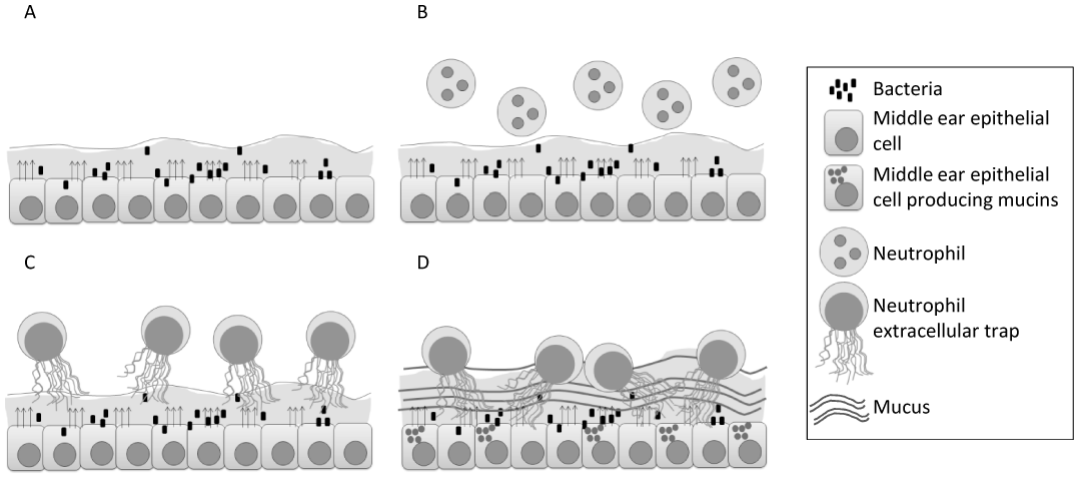
Model for OM progression. Acute bacterial infection (A) leads to the secretion of factors by the middle ear epithelium (shown by arrows), setting up a primarily neutrophilic inflammatory response, predominantly mediated by the chemotaxis effect of IL8 (B). Recruited neutrophils kill bacteria both through phagocytosis and NETosis (C). The resulting infected fluid can persist and recurrent inflammation results in epithelial mucin glycoprotein overproduction and secretion, predominantly MUC5B, which in turn associates with accumulated NETs (D).

Based on our results we hypothesize a model for OM progression whereby acute bacterial infection leads to middle ear epithelial secretion of mediators (Fig 6A), which set up a primarily neutrophilic inflammatory response, predominantly mediated by the chemotaxic effect of IL8 (Fig 6B). Recruited neutrophils can effect bacterial kill both through phagocytosis and NETosis (Fig 6C), the latter ostensibly being triggered by the cytokine itself or bacterial effect on the neutrophil. At this point, the resulting infected fluid will either clear or persist. Persistent or recurrent inflammation results in epithelial mucin glycoprotein overproduction and secretion, predominantly MUC5B, which in turn associates with accumulated NETs (Fig 6D). Indeed NET mediators themselves, such as neutrophil elastase, may promote and sustain epithelial mucin hyper-secretion[57] Middle ear NETs ultimately get involved with bacterial biofilms as host components of the biofilm milieu[22,44] which further potentiates non-clearance of MEE, due to the exceedingly high viscosity of mucin and extracellular DNA combination in the fluid[58]. Of notable interest, the presence of eosinophilic derived DNA extracellular traps in sinonasal secretions has been noted in patients with allergic upper airway diseases, where these traps have been shown to correlate with secretion viscosity and non-clearance[59], effectively an allergic parallel to our posited OM progression model. It is necessary to point out that in our toddler aged OM patient cohort, eosinophils and allergic markers were not noted to be abundant in the MEE proteome (<13 eosinophilic cationic protein peptide counts across samples).

Clearly our report is limited though by the fact that our characterization of mediators in middle ear fluid only represent a “snapshot” in time, and as such the proposed model is only an extrapolation of the potential role of the identified mediators in OM progression over time, and cannot necessarily be directly surmised from the data on hand. Another limitation of our work includes the fact that not every single middle ear fluid specimen could be profiled by each experimental technique. However, we believe the clear uniformity of NET presence in all specimens (stained fresh and immediately to avoid spontaneous production of NETs) by each technique employed demonstrates the importance of NETs in COM. Notably, detection by WB in every single specimen proved to be difficult given the fact that proteins might be degraded by the high abundance of proteases likely coming from neutrophils. As such mass spectrometry techniques, which recognize peptides and not specific epitopes, are more sensitive for protein identification than western blotting and less affected by natural protein degradation during sample procurement and processing. A final limitation of our work is the fact that we did not measure viscosity of the effusions, as such although we surmise that the noted association between NETs and MUC5B is likely to significantly contribute to increased MEE viscosity, we cannot demonstrably conclude as much.

## Conclusion

In conclusion, an unbiased proteomic analysis of MEE revealed that innate immune responses in COME are typically neutrophilic in nature, with NETs being a primary macromolecular constituent of human mucoid middle ear effusions.

## Supporting Information

S1 Table Supplementary data: Proteins detected by MS/MS in mucoid middle ear effusions (MEEs). This table shows the peptide counts of the serum sample and the average peptide count of 9 MEEs for each protein. The cutoff peptide count was set at 2 per MEE.
(DOC)
