# Supplementary material for: Proteomic Characterization of Middle Ear Fluid Confirms Neutrophil Extracellular Traps as a Predominant Innate Immune Response in Chronic Otitis Media
Source: PLoS One. 2016 Apr 14;11(4):e0152865. doi: 10.1371/journal.pone.0152865 (PMC4831838; doi:10.1371/journal.pone.0152865)
Supplement: S1 Table — This table shows the peptide counts of the serum sample and the average peptide count of 9 MEEs for each protein. The cutoff peptide count was set at 2 per MEE. (DOC) [file pone.0152865.s001.doc]

**S1 Table. Supplementary data: Proteins detected by MS/MS in mucoid middle ear effusions (MEEs).**

| **Accession** | **Protein ID** | **Average PC** |
| --- | --- | --- |
| P60709 | Actin, cytoplasmic 1 OS=Homo sapiens GN=ACTB PE=1 SV=1 - [ACTB_HUMAN] | 620 |
| P02788 | Lactotransferrin OS=Homo sapiens GN=LTF PE=1 SV=6 - [TRFL_HUMAN] | 532 |
| Q8TDL5 | BPI fold-containing family B member 1 OS=Homo sapiens GN=BPIFB1 PE=2 SV=1 - [BPIB1_HUMAN] | 482 |
| P35579 | Myosin-9 OS=Homo sapiens GN=MYH9 PE=1 SV=4 - [MYH9_HUMAN] | 454 |
| P68032 | Actin, alpha cardiac muscle 1 OS=Homo sapiens GN=ACTC1 PE=1 SV=1 - [ACTC_HUMAN] | 415 |
| P05164 | Myeloperoxidase OS=Homo sapiens GN=MPO PE=1 SV=1 - [PERM_HUMAN] | 341 |
| P02675 | Fibrinogen beta chain OS=Homo sapiens GN=FGB PE=1 SV=2 - [FIBB_HUMAN] | 221 |
| P13796 | Plastin-2 OS=Homo sapiens GN=LCP1 PE=1 SV=6 - [PLSL_HUMAN] | 217 |
| P68133 | Actin, alpha skeletal muscle OS=Homo sapiens GN=ACTA1 PE=1 SV=1 - [ACTS_HUMAN] | 214 |
| Q9HC84 | Mucin-5B OS=Homo sapiens GN=MUC5B PE=1 SV=3 - [MUC5B_HUMAN] | 211 |
| P06702 | Protein S100-A9 OS=Homo sapiens GN=S100A9 PE=1 SV=1 - [S10A9_HUMAN] | 191 |
| P01876 | Ig alpha-1 chain C region OS=Homo sapiens GN=IGHA1 PE=1 SV=2 - [IGHA1_HUMAN] | 177 |
| P58876 | Histone H2B type 1-D OS=Homo sapiens GN=HIST1H2BD PE=1 SV=2 - [H2B1D_HUMAN] | 177 |
| P06396 | Gelsolin OS=Homo sapiens GN=GSN PE=1 SV=1 - [GELS_HUMAN] | 175 |
| P23527 | Histone H2B type 1-O OS=Homo sapiens GN=HIST1H2BO PE=1 SV=3 - [H2B1O_HUMAN] | 168 |
| P06733 | Alpha-enolase OS=Homo sapiens GN=ENO1 PE=1 SV=2 - [ENOA_HUMAN] | 155 |
| P21333 | Filamin-A OS=Homo sapiens GN=FLNA PE=1 SV=4 - [FLNA_HUMAN] | 154 |
| P30740 | Leukocyte elastase inhibitor OS=Homo sapiens GN=SERPINB1 PE=1 SV=1 - [ILEU_HUMAN] | 153 |
| P14618 | Pyruvate kinase isozymes M1/M2 OS=Homo sapiens GN=PKM PE=1 SV=4 - [KPYM_HUMAN] | 152 |
| P01861 | Ig gamma-4 chain C region OS=Homo sapiens GN=IGHG4 PE=1 SV=1 - [IGHG4_HUMAN] | 151 |
| P12814 | Alpha-actinin-1 OS=Homo sapiens GN=ACTN1 PE=1 SV=2 - [ACTN1_HUMAN] | 148 |
| P08670 | Vimentin OS=Homo sapiens GN=VIM PE=1 SV=4 - [VIME_HUMAN] | 147 |
| P01011 | Alpha-1-antichymotrypsin OS=Homo sapiens GN=SERPINA3 PE=1 SV=2 - [AACT_HUMAN] | 142 |
| P04406 | Glyceraldehyde-3-phosphate dehydrogenase OS=Homo sapiens GN=GAPDH PE=1 SV=3 - [G3P_HUMAN] | 141 |
| P62805 | Histone H4 OS=Homo sapiens GN=HIST1H4A PE=1 SV=2 - [H4_HUMAN] | 138 |
| P04083 | Annexin A1 OS=Homo sapiens GN=ANXA1 PE=1 SV=2 - [ANXA1_HUMAN] | 137 |
| P0CG05 | Ig lambda-2 chain C regions OS=Homo sapiens GN=IGLC2 PE=1 SV=1 - [LAC2_HUMAN] | 137 |
| P02751 | Fibronectin OS=Homo sapiens GN=FN1 PE=1 SV=4 - [FINC_HUMAN] | 136 |
| Q562R1 | Beta-actin-like protein 2 OS=Homo sapiens GN=ACTBL2 PE=1 SV=2 - [ACTBL_HUMAN] | 133 |
| P29401 | Transketolase OS=Homo sapiens GN=TKT PE=1 SV=3 - [TKT_HUMAN] | 128 |
| P26038 | Moesin OS=Homo sapiens GN=MSN PE=1 SV=3 - [MOES_HUMAN] | 127 |
| P11215 | Integrin alpha-M OS=Homo sapiens GN=ITGAM PE=1 SV=2 - [ITAM_HUMAN] | 121 |
| P01833 | Polymeric immunoglobulin receptor OS=Homo sapiens GN=PIGR PE=1 SV=4 - [PIGR_HUMAN] | 113 |
| P08133 | Annexin A6 OS=Homo sapiens GN=ANXA6 PE=1 SV=3 - [ANXA6_HUMAN] | 113 |
| P08311 | Cathepsin G OS=Homo sapiens GN=CTSG PE=1 SV=2 - [CATG_HUMAN] | 108 |
| Q9NP55 | BPI fold-containing family A member 1 OS=Homo sapiens GN=BPIFA1 PE=1 SV=1 - [BPIA1_HUMAN] | 104 |
| O43707 | Alpha-actinin-4 OS=Homo sapiens GN=ACTN4 PE=1 SV=2 - [ACTN4_HUMAN] | 98 |
| P01625 | Ig kappa chain V-IV region Len OS=Homo sapiens PE=1 SV=2 - [KV402_HUMAN] | 97 |
| P12429 | Annexin A3 OS=Homo sapiens GN=ANXA3 PE=1 SV=3 - [ANXA3_HUMAN] | 97 |
| P11142 | Heat shock cognate 71 kDa protein OS=Homo sapiens GN=HSPA8 PE=1 SV=1 - [HSP7C_HUMAN] | 96 |
| P00751 | Complement factor B OS=Homo sapiens GN=CFB PE=1 SV=2 - [CFAB_HUMAN] | 92 |
| P08107 | Heat shock 70 kDa protein 1A/1B OS=Homo sapiens GN=HSPA1A PE=1 SV=5 - [HSP71_HUMAN] | 90 |
| P04040 | Catalase OS=Homo sapiens GN=CAT PE=1 SV=3 - [CATA_HUMAN] | 82 |
| P06744 | Glucose-6-phosphate isomerase OS=Homo sapiens GN=GPI PE=1 SV=4 - [G6PI_HUMAN] | 82 |
| P14780 | Matrix metalloproteinase-9 OS=Homo sapiens GN=MMP9 PE=1 SV=3 - [MMP9_HUMAN] | 78 |
| P08246 | Neutrophil elastase OS=Homo sapiens GN=ELANE PE=1 SV=1 - [ELNE_HUMAN] | 76 |
| P00558 | Phosphoglycerate kinase 1 OS=Homo sapiens GN=PGK1 PE=1 SV=3 - [PGK1_HUMAN] | 75 |
| P18136 | Ig kappa chain V-III region HIC OS=Homo sapiens PE=2 SV=2 - [KV313_HUMAN] | 75 |
| P11021 | 78 kDa glucose-regulated protein OS=Homo sapiens GN=HSPA5 PE=1 SV=2 - [GRP78_HUMAN] | 72 |
| P07900 | Heat shock protein HSP 90-alpha OS=Homo sapiens GN=HSP90AA1 PE=1 SV=5 - [HS90A_HUMAN] | 67 |
| P08238 | Heat shock protein HSP 90-beta OS=Homo sapiens GN=HSP90AB1 PE=1 SV=4 - [HS90B_HUMAN] | 65 |
| P34931 | Heat shock 70 kDa protein 1-like OS=Homo sapiens GN=HSPA1L PE=1 SV=2 - [HS71L_HUMAN] | 65 |
| P17213 | Bactericidal permeability-increasing protein OS=Homo sapiens GN=BPI PE=1 SV=4 - [BPI_HUMAN] | 62 |
| P06312 | Ig kappa chain V-IV region (Fragment) OS=Homo sapiens GN=IGKV4-1 PE=4 SV=1 - [KV401_HUMAN] | 62 |
| P63104 | 14-3-3 protein zeta/delta OS=Homo sapiens GN=YWHAZ PE=1 SV=1 - [1433Z_HUMAN] | 61 |
| P01766 | Ig heavy chain V-III region BRO OS=Homo sapiens PE=1 SV=1 - [HV305_HUMAN] | 59 |
| P07437 | Tubulin beta chain OS=Homo sapiens GN=TUBB PE=1 SV=2 - [TBB5_HUMAN] | 58 |
| Q9BQE3 | Tubulin alpha-1C chain OS=Homo sapiens GN=TUBA1C PE=1 SV=1 - [TBA1C_HUMAN] | 56 |
| P24158 | Myeloblastin OS=Homo sapiens GN=PRTN3 PE=1 SV=3 - [PRTN3_HUMAN] | 55 |
| P27105 | Erythrocyte band 7 integral membrane protein OS=Homo sapiens GN=STOM PE=1 SV=3 - [STOM_HUMAN] | 54 |
| P07355 | Annexin A2 OS=Homo sapiens GN=ANXA2 PE=1 SV=2 - [ANXA2_HUMAN] | 53 |
| Q13885 | Tubulin beta-2A chain OS=Homo sapiens GN=TUBB2A PE=1 SV=1 - [TBB2A_HUMAN] | 52 |
| P52209 | 6-phosphogluconate dehydrogenase, decarboxylating OS=Homo sapiens GN=PGD PE=1 SV=3 - [6PGD_HUMAN] | 52 |
| P00338 | L-lactate dehydrogenase A chain OS=Homo sapiens GN=LDHA PE=1 SV=2 - [LDHA_HUMAN] | 51 |
| P00915 | Carbonic anhydrase 1 OS=Homo sapiens GN=CA1 PE=1 SV=2 - [CAH1_HUMAN] | 51 |
| P16104 | Histone H2A.x OS=Homo sapiens GN=H2AFX PE=1 SV=2 - [H2AX_HUMAN] | 51 |
| P40121 | Macrophage-capping protein OS=Homo sapiens GN=CAPG PE=1 SV=2 - [CAPG_HUMAN] | 51 |
| P68371 | Tubulin beta-4B chain OS=Homo sapiens GN=TUBB4B PE=1 SV=1 - [TBB4B_HUMAN] | 50 |
| P04207 | Ig kappa chain V-III region CLL OS=Homo sapiens PE=1 SV=2 - [KV308_HUMAN] | 50 |
| P04075 | Fructose-bisphosphate aldolase A OS=Homo sapiens GN=ALDOA PE=1 SV=2 - [ALDOA_HUMAN] | 49 |
| P46940 | Ras GTPase-activating-like protein IQGAP1 OS=Homo sapiens GN=IQGAP1 PE=1 SV=1 - [IQGA1_HUMAN] | 47 |
| Q9Y490 | Talin-1 OS=Homo sapiens GN=TLN1 PE=1 SV=3 - [TLN1_HUMAN] | 47 |
| P80188 | Neutrophil gelatinase-associated lipocalin OS=Homo sapiens GN=LCN2 PE=1 SV=2 - [NGAL_HUMAN] | 47 |
| P07737 | Profilin-1 OS=Homo sapiens GN=PFN1 PE=1 SV=2 - [PROF1_HUMAN] | 46 |
| P69891 | Hemoglobin subunit gamma-1 OS=Homo sapiens GN=HBG1 PE=1 SV=2 - [HBG1_HUMAN] | 46 |
| P41218 | Myeloid cell nuclear differentiation antigen OS=Homo sapiens GN=MNDA PE=1 SV=1 - [MNDA_HUMAN] | 43 |
| P09960 | Leukotriene A-4 hydrolase OS=Homo sapiens GN=LTA4H PE=1 SV=2 - [LKHA4_HUMAN] | 43 |
| P22314 | Ubiquitin-like modifier-activating enzyme 1 OS=Homo sapiens GN=UBA1 PE=1 SV=3 - [UBA1_HUMAN] | 43 |
| P31146 | Coronin-1A OS=Homo sapiens GN=CORO1A PE=1 SV=4 - [COR1A_HUMAN] | 42 |
| Q00610 | Clathrin heavy chain 1 OS=Homo sapiens GN=CLTC PE=1 SV=5 - [CLH1_HUMAN] | 42 |
| Q16777 | Histone H2A type 2-C OS=Homo sapiens GN=HIST2H2AC PE=1 SV=4 - [H2A2C_HUMAN] | 42 |
| P07237 | Protein disulfide-isomerase OS=Homo sapiens GN=P4HB PE=1 SV=3 - [PDIA1_HUMAN] | 40 |
| P68104 | Elongation factor 1-alpha 1 OS=Homo sapiens GN=EEF1A1 PE=1 SV=1 - [EF1A1_HUMAN] | 40 |
| Q96KK5 | Histone H2A type 1-H OS=Homo sapiens GN=HIST1H2AH PE=1 SV=3 - [H2A1H_HUMAN] | 40 |
| P15311 | Ezrin OS=Homo sapiens GN=EZR PE=1 SV=4 - [EZRI_HUMAN] | 40 |
| P14625 | Endoplasmin OS=Homo sapiens GN=HSP90B1 PE=1 SV=1 - [ENPL_HUMAN] | 39 |
| P06737 | Glycogen phosphorylase, liver form OS=Homo sapiens GN=PYGL PE=1 SV=4 - [PYGL_HUMAN] | 38 |
| P60174 | Triosephosphate isomerase OS=Homo sapiens GN=TPI1 PE=1 SV=3 - [TPIS_HUMAN] | 38 |
| P08758 | Annexin A5 OS=Homo sapiens GN=ANXA5 PE=1 SV=2 - [ANXA5_HUMAN] | 38 |
| O60814 | Histone H2B type 1-K OS=Homo sapiens GN=HIST1H2BK PE=1 SV=3 - [H2B1K_HUMAN] | 38 |
| P22894 | Neutrophil collagenase OS=Homo sapiens GN=MMP8 PE=1 SV=1 - [MMP8_HUMAN] | 37 |
| P40199 | Carcinoembryonic antigen-related cell adhesion molecule 6 OS=Homo sapiens GN=CEACAM6 PE=1 SV=3 - [CEAM6_HUMAN] | 37 |
| P52566 | Rho GDP-dissociation inhibitor 2 OS=Homo sapiens GN=ARHGDIB PE=1 SV=3 - [GDIR2_HUMAN] | 36 |
| P31946 | 14-3-3 protein beta/alpha OS=Homo sapiens GN=YWHAB PE=1 SV=3 - [1433B_HUMAN] | 35 |
| P06899 | Histone H2B type 1-J OS=Homo sapiens GN=HIST1H2BJ PE=1 SV=3 - [H2B1J_HUMAN] | 35 |
| Q99536 | Synaptic vesicle membrane protein VAT-1 homolog OS=Homo sapiens GN=VAT1 PE=1 SV=2 - [VAT1_HUMAN] | 34 |
| P20160 | Azurocidin OS=Homo sapiens GN=AZU1 PE=1 SV=3 - [CAP7_HUMAN] | 33 |
| P06576 | ATP synthase subunit beta, mitochondrial OS=Homo sapiens GN=ATP5B PE=1 SV=3 - [ATPB_HUMAN] | 33 |
| P35241 | Radixin OS=Homo sapiens GN=RDX PE=1 SV=1 - [RADI_HUMAN] | 32 |
| P52790 | Hexokinase-3 OS=Homo sapiens GN=HK3 PE=1 SV=2 - [HXK3_HUMAN] | 32 |
| P04899 | Guanine nucleotide-binding protein G(i) subunit alpha-2 OS=Homo sapiens GN=GNAI2 PE=1 SV=3 - [GNAI2_HUMAN] | 31 |
| P09467 | Fructose-1,6-bisphosphatase 1 OS=Homo sapiens GN=FBP1 PE=1 SV=5 - [F16P1_HUMAN] | 31 |
| P80723 | Brain acid soluble protein 1 OS=Homo sapiens GN=BASP1 PE=1 SV=2 - [BASP1_HUMAN] | 31 |
| P04839 | Cytochrome b-245 heavy chain OS=Homo sapiens GN=CYBB PE=1 SV=2 - [CY24B_HUMAN] | 30 |
| Q01518 | Adenylyl cyclase-associated protein 1 OS=Homo sapiens GN=CAP1 PE=1 SV=5 - [CAP1_HUMAN] | 30 |
| P23528 | Cofilin-1 OS=Homo sapiens GN=CFL1 PE=1 SV=3 - [COF1_HUMAN] | 30 |
| P61160 | Actin-related protein 2 OS=Homo sapiens GN=ACTR2 PE=1 SV=1 - [ARP2_HUMAN] | 30 |
| Q9UGM3 | Deleted in malignant brain tumors 1 protein OS=Homo sapiens GN=DMBT1 PE=1 SV=2 - [DMBT1_HUMAN] | 30 |
| P11413 | Glucose-6-phosphate 1-dehydrogenase OS=Homo sapiens GN=G6PD PE=1 SV=4 - [G6PD_HUMAN] | 29 |
| Q9Y6R7 | IgGFc-binding protein OS=Homo sapiens GN=FCGBP PE=1 SV=3 - [FCGBP_HUMAN] | 29 |
| P09211 | Glutathione S-transferase P OS=Homo sapiens GN=GSTP1 PE=1 SV=2 - [GSTP1_HUMAN] | 29 |
| P68366 | Tubulin alpha-4A chain OS=Homo sapiens GN=TUBA4A PE=1 SV=1 - [TBA4A_HUMAN] | 29 |
| P61158 | Actin-related protein 3 OS=Homo sapiens GN=ACTR3 PE=1 SV=3 - [ARP3_HUMAN] | 29 |
| P18206 | Vinculin OS=Homo sapiens GN=VCL PE=1 SV=4 - [VINC_HUMAN] | 28 |
| P30101 | Protein disulfide-isomerase A3 OS=Homo sapiens GN=PDIA3 PE=1 SV=4 - [PDIA3_HUMAN] | 28 |
| P18669 | Phosphoglycerate mutase 1 OS=Homo sapiens GN=PGAM1 PE=1 SV=2 - [PGAM1_HUMAN] | 28 |
| P05107 | Integrin beta-2 OS=Homo sapiens GN=ITGB2 PE=1 SV=2 - [ITB2_HUMAN] | 28 |
| P28676 | Grancalcin OS=Homo sapiens GN=GCA PE=1 SV=2 - [GRAN_HUMAN] | 28 |
| P43490 | Nicotinamide phosphoribosyltransferase OS=Homo sapiens GN=NAMPT PE=1 SV=1 - [NAMPT_HUMAN] | 27 |
| P31997 | Carcinoembryonic antigen-related cell adhesion molecule 8 OS=Homo sapiens GN=CEACAM8 PE=1 SV=2 - [CEAM8_HUMAN] | 26 |
| P37837 | Transaldolase OS=Homo sapiens GN=TALDO1 PE=1 SV=2 - [TALDO_HUMAN] | 26 |
| Q9UM07 | Protein-arginine deiminase type-4 OS=Homo sapiens GN=PADI4 PE=1 SV=2 - [PADI4_HUMAN] | 26 |
| P02786 | Transferrin receptor protein 1 OS=Homo sapiens GN=TFRC PE=1 SV=2 - [TFR1_HUMAN] | 26 |
| P01781 | Ig heavy chain V-III region GAL OS=Homo sapiens PE=1 SV=1 - [HV320_HUMAN] | 26 |
| P08575 | Receptor-type tyrosine-protein phosphatase C OS=Homo sapiens GN=PTPRC PE=1 SV=2 - [PTPRC_HUMAN] | 25 |
| P01880 | Ig delta chain C region OS=Homo sapiens GN=IGHD PE=1 SV=2 - [IGHD_HUMAN] | 25 |
| Q6UX06 | Olfactomedin-4 OS=Homo sapiens GN=OLFM4 PE=1 SV=1 - [OLFM4_HUMAN] | 24 |
| P80748 | Ig lambda chain V-III region LOI OS=Homo sapiens PE=1 SV=1 - [LV302_HUMAN] | 24 |
| O15144 | Actin-related protein 2/3 complex subunit 2 OS=Homo sapiens GN=ARPC2 PE=1 SV=1 - [ARPC2_HUMAN] | 23 |
| P61626 | Lysozyme C OS=Homo sapiens GN=LYZ PE=1 SV=1 - [LYSC_HUMAN] | 23 |
| P06753 | Tropomyosin alpha-3 chain OS=Homo sapiens GN=TPM3 PE=1 SV=2 - [TPM3_HUMAN] | 23 |
| P78324 | Tyrosine-protein phosphatase non-receptor type substrate 1 OS=Homo sapiens GN=SIRPA PE=1 SV=2 - [SHPS1_HUMAN] | 22 |
| Q15149 | Plectin OS=Homo sapiens GN=PLEC PE=1 SV=3 - [PLEC_HUMAN] | 22 |
| P04004 | Vitronectin OS=Homo sapiens GN=VTN PE=1 SV=1 - [VTNC_HUMAN] | 22 |
| P51884 | Lumican OS=Homo sapiens GN=LUM PE=1 SV=2 - [LUM_HUMAN] | 22 |
| P01608 | Ig kappa chain V-I region Roy OS=Homo sapiens PE=1 SV=1 - [KV116_HUMAN] | 21 |
| P50395 | Rab GDP dissociation inhibitor beta OS=Homo sapiens GN=GDI2 PE=1 SV=2 - [GDIB_HUMAN] | 21 |
| Q86UX7 | Fermitin family homolog 3 OS=Homo sapiens GN=FERMT3 PE=1 SV=1 - [URP2_HUMAN] | 20 |
| P36222 | Chitinase-3-like protein 1 OS=Homo sapiens GN=CHI3L1 PE=1 SV=2 - [CH3L1_HUMAN] | 20 |
| Q12913 | Receptor-type tyrosine-protein phosphatase eta OS=Homo sapiens GN=PTPRJ PE=1 SV=3 - [PTPRJ_HUMAN] | 20 |
| P29508 | Serpin B3 OS=Homo sapiens GN=SERPINB3 PE=1 SV=2 - [SPB3_HUMAN] | 20 |
| P27824 | Calnexin OS=Homo sapiens GN=CANX PE=1 SV=2 - [CALX_HUMAN] | 19 |
| P62258 | 14-3-3 protein epsilon OS=Homo sapiens GN=YWHAE PE=1 SV=1 - [1433E_HUMAN] | 19 |
| P51149 | Ras-related protein Rab-7a OS=Homo sapiens GN=RAB7A PE=1 SV=1 - [RAB7A_HUMAN] | 19 |
| P20061 | Transcobalamin-1 OS=Homo sapiens GN=TCN1 PE=1 SV=2 - [TCO1_HUMAN] | 19 |
| P0CG48 | Polyubiquitin-C OS=Homo sapiens GN=UBC PE=1 SV=3 - [UBC_HUMAN] | 19 |
| P26641 | Elongation factor 1-gamma OS=Homo sapiens GN=EEF1G PE=1 SV=3 - [EF1G_HUMAN] | 19 |
| P19105 | Myosin regulatory light chain 12A OS=Homo sapiens GN=MYL12A PE=1 SV=2 - [ML12A_HUMAN] | 19 |
| P17931 | Galectin-3 OS=Homo sapiens GN=LGALS3 PE=1 SV=5 - [LEG3_HUMAN] | 19 |
| P27348 | 14-3-3 protein theta OS=Homo sapiens GN=YWHAQ PE=1 SV=1 - [1433T_HUMAN] | 19 |
| P08185 | Corticosteroid-binding globulin OS=Homo sapiens GN=SERPINA6 PE=1 SV=1 - [CBG_HUMAN] | 18 |
| P10412 | Histone H1.4 OS=Homo sapiens GN=HIST1H1E PE=1 SV=2 - [H14_HUMAN] | 18 |
| P50453 | Serpin B9 OS=Homo sapiens GN=SERPINB9 PE=1 SV=1 - [SPB9_HUMAN] | 18 |
| P09525 | Annexin A4 OS=Homo sapiens GN=ANXA4 PE=1 SV=4 - [ANXA4_HUMAN] | 18 |
| P07195 | L-lactate dehydrogenase B chain OS=Homo sapiens GN=LDHB PE=1 SV=2 - [LDHB_HUMAN] | 17 |
| P27797 | Calreticulin OS=Homo sapiens GN=CALR PE=1 SV=1 - [CALR_HUMAN] | 17 |
| O15143 | Actin-related protein 2/3 complex subunit 1B OS=Homo sapiens GN=ARPC1B PE=1 SV=3 - [ARC1B_HUMAN] | 17 |
| P67936 | Tropomyosin alpha-4 chain OS=Homo sapiens GN=TPM4 PE=1 SV=3 - [TPM4_HUMAN] | 17 |
| P15153 | Ras-related C3 botulinum toxin substrate 2 OS=Homo sapiens GN=RAC2 PE=1 SV=1 - [RAC2_HUMAN] | 17 |
| O15145 | Actin-related protein 2/3 complex subunit 3 OS=Homo sapiens GN=ARPC3 PE=1 SV=3 - [ARPC3_HUMAN] | 17 |
| O00299 | Chloride intracellular channel protein 1 OS=Homo sapiens GN=CLIC1 PE=1 SV=4 - [CLIC1_HUMAN] | 17 |
| P52907 | F-actin-capping protein subunit alpha-1 OS=Homo sapiens GN=CAPZA1 PE=1 SV=3 - [CAZA1_HUMAN] | 17 |
| P20702 | Integrin alpha-X OS=Homo sapiens GN=ITGAX PE=1 SV=3 - [ITAX_HUMAN] | 17 |
| O75083 | WD repeat-containing protein 1 OS=Homo sapiens GN=WDR1 PE=1 SV=4 - [WDR1_HUMAN] | 16 |
| Q13813 | Spectrin alpha chain, non-erythrocytic 1 OS=Homo sapiens GN=SPTAN1 PE=1 SV=3 - [SPTN1_HUMAN] | 16 |
| P05109 | Protein S100-A8 OS=Homo sapiens GN=S100A8 PE=1 SV=1 - [S10A8_HUMAN] | 16 |
| P06727 | Apolipoprotein A-IV OS=Homo sapiens GN=APOA4 PE=1 SV=3 - [APOA4_HUMAN] | 16 |
| P62158 | Calmodulin OS=Homo sapiens GN=CALM1 PE=1 SV=2 - [CALM_HUMAN] | 16 |
| Q9HDC9 | Adipocyte plasma membrane-associated protein OS=Homo sapiens GN=APMAP PE=1 SV=2 - [APMAP_HUMAN] | 16 |
| P60660 | Myosin light polypeptide 6 OS=Homo sapiens GN=MYL6 PE=1 SV=2 - [MYL6_HUMAN] | 16 |
| P04179 | Superoxide dismutase [Mn], mitochondrial OS=Homo sapiens GN=SOD2 PE=1 SV=2 - [SODM_HUMAN] | 15 |
| P19971 | Thymidine phosphorylase OS=Homo sapiens GN=TYMP PE=1 SV=2 - [TYPH_HUMAN] | 15 |
| P61981 | 14-3-3 protein gamma OS=Homo sapiens GN=YWHAG PE=1 SV=2 - [1433G_HUMAN] | 15 |
| P25705 | ATP synthase subunit alpha, mitochondrial OS=Homo sapiens GN=ATP5A1 PE=1 SV=1 - [ATPA_HUMAN] | 15 |
| P63000 | Ras-related C3 botulinum toxin substrate 1 OS=Homo sapiens GN=RAC1 PE=1 SV=1 - [RAC1_HUMAN] | 15 |
| Q9H0U4 | Ras-related protein Rab-1B OS=Homo sapiens GN=RAB1B PE=1 SV=1 - [RAB1B_HUMAN] | 15 |
| P02792 | Ferritin light chain OS=Homo sapiens GN=FTL PE=1 SV=2 - [FRIL_HUMAN] | 15 |
| Q9NUQ9 | Protein FAM49B OS=Homo sapiens GN=FAM49B PE=1 SV=1 - [FA49B_HUMAN] | 15 |
| P22626 | Heterogeneous nuclear ribonucleoproteins A2/B1 OS=Homo sapiens GN=HNRNPA2B1 PE=1 SV=2 - [ROA2_HUMAN] | 15 |
| P50995 | Annexin A11 OS=Homo sapiens GN=ANXA11 PE=1 SV=1 - [ANX11_HUMAN] | 14 |
| P13688 | Carcinoembryonic antigen-related cell adhesion molecule 1 OS=Homo sapiens GN=CEACAM1 PE=1 SV=2 - [CEAM1_HUMAN] | 14 |
| P61026 | Ras-related protein Rab-10 OS=Homo sapiens GN=RAB10 PE=1 SV=1 - [RAB10_HUMAN] | 14 |
| P55072 | Transitional endoplasmic reticulum ATPase OS=Homo sapiens GN=VCP PE=1 SV=4 - [TERA_HUMAN] | 14 |
| P09651 | Heterogeneous nuclear ribonucleoprotein A1 OS=Homo sapiens GN=HNRNPA1 PE=1 SV=5 - [ROA1_HUMAN] | 14 |
| P49913 | Cathelicidin antimicrobial peptide OS=Homo sapiens GN=CAMP PE=1 SV=1 - [CAMP_HUMAN] | 13 |
| P52565 | Rho GDP-dissociation inhibitor 1 OS=Homo sapiens GN=ARHGDIA PE=1 SV=3 - [GDIR1_HUMAN] | 13 |
| O00160 | Unconventional myosin-If OS=Homo sapiens GN=MYO1F PE=1 SV=3 - [MYO1F_HUMAN] | 13 |
| P16190 | HLA class I histocompatibility antigen, A-33 alpha chain OS=Homo sapiens GN=HLA-A PE=2 SV=3 - [1A33_HUMAN] | 13 |
| P30464 | HLA class I histocompatibility antigen, B-15 alpha chain OS=Homo sapiens GN=HLA-B PE=1 SV=2 - [1B15_HUMAN] | 13 |
| P29966 | Myristoylated alanine-rich C-kinase substrate OS=Homo sapiens GN=MARCKS PE=1 SV=4 - [MARCS_HUMAN] | 13 |
| P30044 | Peroxiredoxin-5, mitochondrial OS=Homo sapiens GN=PRDX5 PE=1 SV=4 - [PRDX5_HUMAN] | 13 |
| Q04917 | 14-3-3 protein eta OS=Homo sapiens GN=YWHAH PE=1 SV=4 - [1433F_HUMAN] | 12 |
| P02794 | Ferritin heavy chain OS=Homo sapiens GN=FTH1 PE=1 SV=2 - [FRIH_HUMAN] | 12 |
| P80511 | Protein S100-A12 OS=Homo sapiens GN=S100A12 PE=1 SV=2 - [S10AC_HUMAN] | 12 |
| P47756 | F-actin-capping protein subunit beta OS=Homo sapiens GN=CAPZB PE=1 SV=4 - [CAPZB_HUMAN] | 12 |
| A6NL28 | Putative tropomyosin alpha-3 chain-like protein OS=Homo sapiens PE=5 SV=2 - [TPM3L_HUMAN] | 12 |
| P00491 | Purine nucleoside phosphorylase OS=Homo sapiens GN=PNP PE=1 SV=2 - [PNPH_HUMAN] | 12 |
| P16188 | HLA class I histocompatibility antigen, A-30 alpha chain OS=Homo sapiens GN=HLA-A PE=2 SV=2 - [1A30_HUMAN] | 12 |
| P30460 | HLA class I histocompatibility antigen, B-8 alpha chain OS=Homo sapiens GN=HLA-B PE=1 SV=1 - [1B08_HUMAN] | 12 |
| Q16555 | Dihydropyrimidinase-related protein 2 OS=Homo sapiens GN=DPYSL2 PE=1 SV=1 - [DPYL2_HUMAN] | 12 |
| Q92930 | Ras-related protein Rab-8B OS=Homo sapiens GN=RAB8B PE=1 SV=2 - [RAB8B_HUMAN] | 12 |
| P23284 | Peptidyl-prolyl cis-trans isomerase B OS=Homo sapiens GN=PPIB PE=1 SV=2 - [PPIB_HUMAN] | 12 |
| P61978 | Heterogeneous nuclear ribonucleoprotein K OS=Homo sapiens GN=HNRNPK PE=1 SV=1 - [HNRPK_HUMAN] | 12 |
| P10643 | Complement component C7 OS=Homo sapiens GN=C7 PE=1 SV=2 - [CO7_HUMAN] | 12 |
| P37802 | Transgelin-2 OS=Homo sapiens GN=TAGLN2 PE=1 SV=3 - [TAGL2_HUMAN] | 11 |
| P20700 | Lamin-B1 OS=Homo sapiens GN=LMNB1 PE=1 SV=2 - [LMNB1_HUMAN] | 11 |
| P22392 | Nucleoside diphosphate kinase B OS=Homo sapiens GN=NME2 PE=1 SV=1 - [NDKB_HUMAN] | 11 |
| P31150 | Rab GDP dissociation inhibitor alpha OS=Homo sapiens GN=GDI1 PE=1 SV=2 - [GDIA_HUMAN] | 11 |
| O75369 | Filamin-B OS=Homo sapiens GN=FLNB PE=1 SV=2 - [FLNB_HUMAN] | 11 |
| P12236 | ADP/ATP translocase 3 OS=Homo sapiens GN=SLC25A6 PE=1 SV=4 - [ADT3_HUMAN] | 11 |
| P19367 | Hexokinase-1 OS=Homo sapiens GN=HK1 PE=1 SV=3 - [HXK1_HUMAN] | 11 |
| P62491 | Ras-related protein Rab-11A OS=Homo sapiens GN=RAB11A PE=1 SV=3 - [RB11A_HUMAN] | 11 |
| Q14697 | Neutral alpha-glucosidase AB OS=Homo sapiens GN=GANAB PE=1 SV=3 - [GANAB_HUMAN] | 11 |
| P07339 | Cathepsin D OS=Homo sapiens GN=CTSD PE=1 SV=1 - [CATD_HUMAN] | 11 |
| Q01082 | Spectrin beta chain, non-erythrocytic 1 OS=Homo sapiens GN=SPTBN1 PE=1 SV=2 - [SPTB2_HUMAN] | 11 |
| P01031 | Complement C5 OS=Homo sapiens GN=C5 PE=1 SV=4 - [CO5_HUMAN] | 11 |
| O75367 | Core histone macro-H2A.1 OS=Homo sapiens GN=H2AFY PE=1 SV=4 - [H2AY_HUMAN] | 10 |
| P0C0S5 | Histone H2A.Z OS=Homo sapiens GN=H2AFZ PE=1 SV=2 - [H2AZ_HUMAN] | 10 |
| P23381 | Tryptophan--tRNA ligase, cytoplasmic OS=Homo sapiens GN=WARS PE=1 SV=2 - [SYWC_HUMAN] | 10 |
| P31943 | Heterogeneous nuclear ribonucleoprotein H OS=Homo sapiens GN=HNRNPH1 PE=1 SV=4 - [HNRH1_HUMAN] | 10 |
| P61586 | Transforming protein RhoA OS=Homo sapiens GN=RHOA PE=1 SV=1 - [RHOA_HUMAN] | 10 |
| P61224 | Ras-related protein Rap-1b OS=Homo sapiens GN=RAP1B PE=1 SV=1 - [RAP1B_HUMAN] | 10 |
| P30041 | Peroxiredoxin-6 OS=Homo sapiens GN=PRDX6 PE=1 SV=3 - [PRDX6_HUMAN] | 10 |
| P12273 | Prolactin-inducible protein OS=Homo sapiens GN=PIP PE=1 SV=1 - [PIP_HUMAN] | 10 |
| P13498 | Cytochrome b-245 light chain OS=Homo sapiens GN=CYBA PE=1 SV=3 - [CY24A_HUMAN] | 10 |
| P52597 | Heterogeneous nuclear ribonucleoprotein F OS=Homo sapiens GN=HNRNPF PE=1 SV=3 - [HNRPF_HUMAN] | 10 |
| Q99714 | 3-hydroxyacyl-CoA dehydrogenase type-2 OS=Homo sapiens GN=HSD17B10 PE=1 SV=3 - [HCD2_HUMAN] | 10 |
| P12724 | Eosinophil cationic protein OS=Homo sapiens GN=RNASE3 PE=1 SV=2 - [ECP_HUMAN] | 10 |
| P62937 | Peptidyl-prolyl cis-trans isomerase A OS=Homo sapiens GN=PPIA PE=1 SV=2 - [PPIA_HUMAN] | 10 |
| P62820 | Ras-related protein Rab-1A OS=Homo sapiens GN=RAB1A PE=1 SV=3 - [RAB1A_HUMAN] | 10 |
| Q15907 | Ras-related protein Rab-11B OS=Homo sapiens GN=RAB11B PE=1 SV=4 - [RB11B_HUMAN] | 10 |
| O14818 | Proteasome subunit alpha type-7 OS=Homo sapiens GN=PSMA7 PE=1 SV=1 - [PSA7_HUMAN] | 10 |
| Q06830 | Peroxiredoxin-1 OS=Homo sapiens GN=PRDX1 PE=1 SV=1 - [PRDX1_HUMAN] | 10 |
| P16401 | Histone H1.5 OS=Homo sapiens GN=HIST1H1B PE=1 SV=3 - [H15_HUMAN] | 10 |
| P14598 | Neutrophil cytosol factor 1 OS=Homo sapiens GN=NCF1 PE=1 SV=3 - [NCF1_HUMAN] | 9 |
| P60900 | Proteasome subunit alpha type-6 OS=Homo sapiens GN=PSMA6 PE=1 SV=1 - [PSA6_HUMAN] | 9 |
| P00403 | Cytochrome c oxidase subunit 2 OS=Homo sapiens GN=MT-CO2 PE=1 SV=1 - [COX2_HUMAN] | 9 |
| P04434 | Ig kappa chain V-III region VH (Fragment) OS=Homo sapiens PE=4 SV=1 - [KV310_HUMAN] | 9 |
| P08754 | Guanine nucleotide-binding protein G(k) subunit alpha OS=Homo sapiens GN=GNAI3 PE=1 SV=3 - [GNAI3_HUMAN] | 9 |
| P11279 | Lysosome-associated membrane glycoprotein 1 OS=Homo sapiens GN=LAMP1 PE=1 SV=3 - [LAMP1_HUMAN] | 9 |
| P61020 | Ras-related protein Rab-5B OS=Homo sapiens GN=RAB5B PE=1 SV=1 - [RAB5B_HUMAN] | 9 |
| P62834 | Ras-related protein Rap-1A OS=Homo sapiens GN=RAP1A PE=1 SV=1 - [RAP1A_HUMAN] | 9 |
| P84085 | ADP-ribosylation factor 5 OS=Homo sapiens GN=ARF5 PE=1 SV=2 - [ARF5_HUMAN] | 9 |
| Q96DA0 | Zymogen granule protein 16 homolog B OS=Homo sapiens GN=ZG16B PE=1 SV=3 - [ZG16B_HUMAN] | 9 |
| P28838 | Cytosol aminopeptidase OS=Homo sapiens GN=LAP3 PE=1 SV=3 - [AMPL_HUMAN] | 9 |
| P13667 | Protein disulfide-isomerase A4 OS=Homo sapiens GN=PDIA4 PE=1 SV=2 - [PDIA4_HUMAN] | 9 |
| Q13231 | Chitotriosidase-1 OS=Homo sapiens GN=CHIT1 PE=1 SV=1 - [CHIT1_HUMAN] | 9 |
| Q15084 | Protein disulfide-isomerase A6 OS=Homo sapiens GN=PDIA6 PE=1 SV=1 - [PDIA6_HUMAN] | 9 |
| P61204 | ADP-ribosylation factor 3 OS=Homo sapiens GN=ARF3 PE=1 SV=2 - [ARF3_HUMAN] | 9 |
| P04208 | Ig lambda chain V-I region WAH OS=Homo sapiens PE=1 SV=1 - [LV106_HUMAN] | 9 |
| P60953 | Cell division control protein 42 homolog OS=Homo sapiens GN=CDC42 PE=1 SV=2 - [CDC42_HUMAN] | 9 |
| P61106 | Ras-related protein Rab-14 OS=Homo sapiens GN=RAB14 PE=1 SV=4 - [RAB14_HUMAN] | 9 |
| P32942 | Intercellular adhesion molecule 3 OS=Homo sapiens GN=ICAM3 PE=1 SV=2 - [ICAM3_HUMAN] | 9 |
| P54819 | Adenylate kinase 2, mitochondrial OS=Homo sapiens GN=AK2 PE=1 SV=2 - [KAD2_HUMAN] | 9 |
| Q96QK1 | Vacuolar protein sorting-associated protein 35 OS=Homo sapiens GN=VPS35 PE=1 SV=2 - [VPS35_HUMAN] | 9 |
| P48595 | Serpin B10 OS=Homo sapiens GN=SERPINB10 PE=1 SV=1 - [SPB10_HUMAN] | 9 |
| P01714 | Ig lambda chain V-III region SH OS=Homo sapiens PE=1 SV=1 - [LV301_HUMAN] | 9 |
| P01721 | Ig lambda chain V-VI region AR OS=Homo sapiens PE=1 SV=1 - [LV601_HUMAN] | 9 |
| O75874 | Isocitrate dehydrogenase [NADP] cytoplasmic OS=Homo sapiens GN=IDH1 PE=1 SV=2 - [IDHC_HUMAN] | 8 |
| P26583 | High mobility group protein B2 OS=Homo sapiens GN=HMGB2 PE=1 SV=2 - [HMGB2_HUMAN] | 8 |
| P00747 | Plasminogen OS=Homo sapiens GN=PLG PE=1 SV=2 - [PLMN_HUMAN] | 8 |
| P50552 | Vasodilator-stimulated phosphoprotein OS=Homo sapiens GN=VASP PE=1 SV=3 - [VASP_HUMAN] | 8 |
| Q07960 | Rho GTPase-activating protein 1 OS=Homo sapiens GN=ARHGAP1 PE=1 SV=1 - [RHG01_HUMAN] | 8 |
| P05141 | ADP/ATP translocase 2 OS=Homo sapiens GN=SLC25A5 PE=1 SV=7 - [ADT2_HUMAN] | 8 |
| P26447 | Protein S100-A4 OS=Homo sapiens GN=S100A4 PE=1 SV=1 - [S10A4_HUMAN] | 8 |
| P13489 | Ribonuclease inhibitor OS=Homo sapiens GN=RNH1 PE=1 SV=2 - [RINI_HUMAN] | 8 |
| P04003 | C4b-binding protein alpha chain OS=Homo sapiens GN=C4BPA PE=1 SV=2 - [C4BPA_HUMAN] | 8 |
| P22079 | Lactoperoxidase OS=Homo sapiens GN=LPO PE=1 SV=2 - [PERL_HUMAN] | 8 |
| P24539 | ATP synthase subunit b, mitochondrial OS=Homo sapiens GN=ATP5F1 PE=1 SV=2 - [AT5F1_HUMAN] | 8 |
| P25787 | Proteasome subunit alpha type-2 OS=Homo sapiens GN=PSMA2 PE=1 SV=2 - [PSA2_HUMAN] | 8 |
| P35232 | Prohibitin OS=Homo sapiens GN=PHB PE=1 SV=1 - [PHB_HUMAN] | 8 |
| Q16531 | DNA damage-binding protein 1 OS=Homo sapiens GN=DDB1 PE=1 SV=1 - [DDB1_HUMAN] | 8 |
| Q92928 | Putative Ras-related protein Rab-1C OS=Homo sapiens GN=RAB1C PE=5 SV=2 - [RAB1C_HUMAN] | 8 |
| O95336 | 6-phosphogluconolactonase OS=Homo sapiens GN=PGLS PE=1 SV=2 - [6PGL_HUMAN] | 8 |
| P25815 | Protein S100-P OS=Homo sapiens GN=S100P PE=1 SV=2 - [S100P_HUMAN] | 8 |
| P62136 | Serine/threonine-protein phosphatase PP1-alpha catalytic subunit OS=Homo sapiens GN=PPP1CA PE=1 SV=1 - [PP1A_HUMAN] | 8 |
| P00736 | Complement C1r subcomponent OS=Homo sapiens GN=C1R PE=1 SV=2 - [C1R_HUMAN] | 8 |
| P40925 | Malate dehydrogenase, cytoplasmic OS=Homo sapiens GN=MDH1 PE=1 SV=4 - [MDHC_HUMAN] | 8 |
| P43652 | Afamin OS=Homo sapiens GN=AFM PE=1 SV=1 - [AFAM_HUMAN] | 8 |
| P22061 | Protein-L-isoaspartate(D-aspartate) O-methyltransferase OS=Homo sapiens GN=PCMT1 PE=1 SV=4 - [PIMT_HUMAN] | 8 |
| P29350 | Tyrosine-protein phosphatase non-receptor type 6 OS=Homo sapiens GN=PTPN6 PE=1 SV=1 - [PTN6_HUMAN] | 8 |
| Q9H4M9 | EH domain-containing protein 1 OS=Homo sapiens GN=EHD1 PE=1 SV=2 - [EHD1_HUMAN] | 8 |
| Q9UJ70 | N-acetyl-D-glucosamine kinase OS=Homo sapiens GN=NAGK PE=1 SV=4 - [NAGK_HUMAN] | 8 |
| P68431 | Histone H3.1 OS=Homo sapiens GN=HIST1H3A PE=1 SV=2 - [H31_HUMAN] | 7 |
| P55058 | Phospholipid transfer protein OS=Homo sapiens GN=PLTP PE=1 SV=1 - [PLTP_HUMAN] | 7 |
| Q16851 | UTP--glucose-1-phosphate uridylyltransferase OS=Homo sapiens GN=UGP2 PE=1 SV=5 - [UGPA_HUMAN] | 7 |
| P51159 | Ras-related protein Rab-27A OS=Homo sapiens GN=RAB27A PE=1 SV=3 - [RB27A_HUMAN] | 7 |
| P62826 | GTP-binding nuclear protein Ran OS=Homo sapiens GN=RAN PE=1 SV=3 - [RAN_HUMAN] | 7 |
| P04080 | Cystatin-B OS=Homo sapiens GN=CSTB PE=1 SV=2 - [CYTB_HUMAN] | 7 |
| P01743 | Ig heavy chain V-I region HG3 OS=Homo sapiens PE=4 SV=1 - [HV102_HUMAN] | 7 |
| P09917 | Arachidonate 5-lipoxygenase OS=Homo sapiens GN=ALOX5 PE=1 SV=2 - [LOX5_HUMAN] | 7 |
| P38606 | V-type proton ATPase catalytic subunit A OS=Homo sapiens GN=ATP6V1A PE=1 SV=2 - [VATA_HUMAN] | 7 |
| Q92542 | Nicastrin OS=Homo sapiens GN=NCSTN PE=1 SV=2 - [NICA_HUMAN] | 7 |
| Q9UJU6 | Drebrin-like protein OS=Homo sapiens GN=DBNL PE=1 SV=1 - [DBNL_HUMAN] | 7 |
| P15531 | Nucleoside diphosphate kinase A OS=Homo sapiens GN=NME1 PE=1 SV=1 - [NDKA_HUMAN] | 7 |
| P13760 | HLA class II histocompatibility antigen, DRB1-4 beta chain OS=Homo sapiens GN=HLA-DRB1 PE=1 SV=1 - [2B14_HUMAN] | 7 |
| P16152 | Carbonyl reductase [NADPH] 1 OS=Homo sapiens GN=CBR1 PE=1 SV=3 - [CBR1_HUMAN] | 7 |
| P25788 | Proteasome subunit alpha type-3 OS=Homo sapiens GN=PSMA3 PE=1 SV=2 - [PSA3_HUMAN] | 7 |
| P45880 | Voltage-dependent anion-selective channel protein 2 OS=Homo sapiens GN=VDAC2 PE=1 SV=2 - [VDAC2_HUMAN] | 7 |
| Q03591 | Complement factor H-related protein 1 OS=Homo sapiens GN=CFHR1 PE=1 SV=2 - [FHR1_HUMAN] | 7 |
| Q16539 | Mitogen-activated protein kinase 14 OS=Homo sapiens GN=MAPK14 PE=1 SV=3 - [MK14_HUMAN] | 7 |
| Q29865 | HLA class I histocompatibility antigen, Cw-18 alpha chain OS=Homo sapiens GN=HLA-C PE=2 SV=1 - [1C18_HUMAN] | 7 |
| Q8IUE6 | Histone H2A type 2-B OS=Homo sapiens GN=HIST2H2AB PE=1 SV=3 - [H2A2B_HUMAN] | 7 |
| Q99623 | Prohibitin-2 OS=Homo sapiens GN=PHB2 PE=1 SV=2 - [PHB2_HUMAN] | 7 |
| Q99828 | Calcium and integrin-binding protein 1 OS=Homo sapiens GN=CIB1 PE=1 SV=4 - [CIB1_HUMAN] | 7 |
| P08865 | 40S ribosomal protein SA OS=Homo sapiens GN=RPSA PE=1 SV=4 - [RSSA_HUMAN] | 7 |
| P28070 | Proteasome subunit beta type-4 OS=Homo sapiens GN=PSMB4 PE=1 SV=4 - [PSB4_HUMAN] | 7 |
| P14550 | Alcohol dehydrogenase [NADP(+)] OS=Homo sapiens GN=AKR1A1 PE=1 SV=3 - [AK1A1_HUMAN] | 7 |
| P40926 | Malate dehydrogenase, mitochondrial OS=Homo sapiens GN=MDH2 PE=1 SV=3 - [MDHM_HUMAN] | 7 |
| P15144 | Aminopeptidase N OS=Homo sapiens GN=ANPEP PE=1 SV=4 - [AMPN_HUMAN] | 7 |
| P01611 | Ig kappa chain V-I region Wes OS=Homo sapiens PE=1 SV=1 - [KV119_HUMAN] | 7 |
| P07686 | Beta-hexosaminidase subunit beta OS=Homo sapiens GN=HEXB PE=1 SV=3 - [HEXB_HUMAN] | 7 |
| P09972 | Fructose-bisphosphate aldolase C OS=Homo sapiens GN=ALDOC PE=1 SV=2 - [ALDOC_HUMAN] | 7 |
| P18085 | ADP-ribosylation factor 4 OS=Homo sapiens GN=ARF4 PE=1 SV=3 - [ARF4_HUMAN] | 7 |
| Q10588 | ADP-ribosyl cyclase 2 OS=Homo sapiens GN=BST1 PE=1 SV=2 - [BST1_HUMAN] | 7 |
| Q9ULZ3 | Apoptosis-associated speck-like protein containing a CARD OS=Homo sapiens GN=PYCARD PE=1 SV=2 - [ASC_HUMAN] | 7 |
| P05089 | Arginase-1 OS=Homo sapiens GN=ARG1 PE=1 SV=2 - [ARGI1_HUMAN] | 7 |
| Q9H299 | SH3 domain-binding glutamic acid-rich-like protein 3 OS=Homo sapiens GN=SH3BGRL3 PE=1 SV=1 - [SH3L3_HUMAN] | 7 |
| P06748 | Nucleophosmin OS=Homo sapiens GN=NPM1 PE=1 SV=2 - [NPM_HUMAN] | 6 |
| O00602 | Ficolin-1 OS=Homo sapiens GN=FCN1 PE=1 SV=2 - [FCN1_HUMAN] | 6 |
| P61019 | Ras-related protein Rab-2A OS=Homo sapiens GN=RAB2A PE=1 SV=1 - [RAB2A_HUMAN] | 6 |
| P31949 | Protein S100-A11 OS=Homo sapiens GN=S100A11 PE=1 SV=2 - [S10AB_HUMAN] | 6 |
| Q6IBS0 | Twinfilin-2 OS=Homo sapiens GN=TWF2 PE=1 SV=2 - [TWF2_HUMAN] | 6 |
| Q15080 | Neutrophil cytosol factor 4 OS=Homo sapiens GN=NCF4 PE=1 SV=2 - [NCF4_HUMAN] | 6 |
| O75347 | Tubulin-specific chaperone A OS=Homo sapiens GN=TBCA PE=1 SV=3 - [TBCA_HUMAN] | 6 |
| P13671 | Complement component C6 OS=Homo sapiens GN=C6 PE=1 SV=3 - [CO6_HUMAN] | 6 |
| P04229 | HLA class II histocompatibility antigen, DRB1-1 beta chain OS=Homo sapiens GN=HLA-DRB1 PE=1 SV=2 - [2B11_HUMAN] | 6 |
| P05091 | Aldehyde dehydrogenase, mitochondrial OS=Homo sapiens GN=ALDH2 PE=1 SV=2 - [ALDH2_HUMAN] | 6 |
| P05186 | Alkaline phosphatase, tissue-nonspecific isozyme OS=Homo sapiens GN=ALPL PE=1 SV=4 - [PPBT_HUMAN] | 6 |
| P10316 | HLA class I histocompatibility antigen, A-69 alpha chain OS=Homo sapiens GN=HLA-A PE=2 SV=2 - [1A69_HUMAN] | 6 |
| P13761 | HLA class II histocompatibility antigen, DRB1-7 beta chain OS=Homo sapiens GN=HLA-DRB1 PE=1 SV=1 - [2B17_HUMAN] | 6 |
| P16083 | Ribosyldihydronicotinamide dehydrogenase [quinone] OS=Homo sapiens GN=NQO2 PE=1 SV=5 - [NQO2_HUMAN] | 6 |
| P22695 | Cytochrome b-c1 complex subunit 2, mitochondrial OS=Homo sapiens GN=UQCRC2 PE=1 SV=3 - [QCR2_HUMAN] | 6 |
| P28482 | Mitogen-activated protein kinase 1 OS=Homo sapiens GN=MAPK1 PE=1 SV=3 - [MK01_HUMAN] | 6 |
| P30084 | Enoyl-CoA hydratase, mitochondrial OS=Homo sapiens GN=ECHS1 PE=1 SV=4 - [ECHM_HUMAN] | 6 |
| Q14980 | Nuclear mitotic apparatus protein 1 OS=Homo sapiens GN=NUMA1 PE=1 SV=2 - [NUMA1_HUMAN] | 6 |
| Q96FW1 | Ubiquitin thioesterase OTUB1 OS=Homo sapiens GN=OTUB1 PE=1 SV=2 - [OTUB1_HUMAN] | 6 |
| P57737 | Coronin-7 OS=Homo sapiens GN=CORO7 PE=1 SV=2 - [CORO7_HUMAN] | 6 |
| P07384 | Calpain-1 catalytic subunit OS=Homo sapiens GN=CAPN1 PE=1 SV=1 - [CAN1_HUMAN] | 6 |
| O00764 | Pyridoxal kinase OS=Homo sapiens GN=PDXK PE=1 SV=1 - [PDXK_HUMAN] | 6 |
| P59998 | Actin-related protein 2/3 complex subunit 4 OS=Homo sapiens GN=ARPC4 PE=1 SV=3 - [ARPC4_HUMAN] | 6 |
| P20618 | Proteasome subunit beta type-1 OS=Homo sapiens GN=PSMB1 PE=1 SV=2 - [PSB1_HUMAN] | 6 |
| P25786 | Proteasome subunit alpha type-1 OS=Homo sapiens GN=PSMA1 PE=1 SV=1 - [PSA1_HUMAN] | 6 |
| P50990 | T-complex protein 1 subunit theta OS=Homo sapiens GN=CCT8 PE=1 SV=4 - [TCPQ_HUMAN] | 6 |
| Q6P4A8 | Phospholipase B-like 1 OS=Homo sapiens GN=PLBD1 PE=1 SV=2 - [PLBL1_HUMAN] | 6 |
| P24821 | Tenascin OS=Homo sapiens GN=TNC PE=1 SV=3 - [TENA_HUMAN] | 6 |
| O75131 | Copine-3 OS=Homo sapiens GN=CPNE3 PE=1 SV=1 - [CPNE3_HUMAN] | 5 |
| P10153 | Non-secretory ribonuclease OS=Homo sapiens GN=RNASE2 PE=1 SV=2 - [RNAS2_HUMAN] | 5 |
| Q96CX2 | BTB/POZ domain-containing protein KCTD12 OS=Homo sapiens GN=KCTD12 PE=1 SV=1 - [KCD12_HUMAN] | 5 |
| Q99497 | Protein DJ-1 OS=Homo sapiens GN=PARK7 PE=1 SV=2 - [PARK7_HUMAN] | 5 |
| P07910 | Heterogeneous nuclear ribonucleoproteins C1/C2 OS=Homo sapiens GN=HNRNPC PE=1 SV=4 - [HNRPC_HUMAN] | 5 |
| O43451 | Maltase-glucoamylase, intestinal OS=Homo sapiens GN=MGAM PE=1 SV=5 - [MGA_HUMAN] | 5 |
| Q13510 | Acid ceramidase OS=Homo sapiens GN=ASAH1 PE=1 SV=5 - [ASAH1_HUMAN] | 5 |
| O15511 | Actin-related protein 2/3 complex subunit 5 OS=Homo sapiens GN=ARPC5 PE=1 SV=3 - [ARPC5_HUMAN] | 5 |
| P51148 | Ras-related protein Rab-5C OS=Homo sapiens GN=RAB5C PE=1 SV=2 - [RAB5C_HUMAN] | 5 |
| P01613 | Ig kappa chain V-I region Ni OS=Homo sapiens PE=1 SV=1 - [KV121_HUMAN] | 5 |
| O75368 | SH3 domain-binding glutamic acid-rich-like protein OS=Homo sapiens GN=SH3BGRL PE=1 SV=1 - [SH3L1_HUMAN] | 5 |
| P01889 | HLA class I histocompatibility antigen, B-7 alpha chain OS=Homo sapiens GN=HLA-B PE=1 SV=3 - [1B07_HUMAN] | 5 |
| P11234 | Ras-related protein Ral-B OS=Homo sapiens GN=RALB PE=1 SV=1 - [RALB_HUMAN] | 5 |
| P23526 | Adenosylhomocysteinase OS=Homo sapiens GN=AHCY PE=1 SV=4 - [SAHH_HUMAN] | 5 |
| P27695 | DNA-(apurinic or apyrimidinic site) lyase OS=Homo sapiens GN=APEX1 PE=1 SV=2 - [APEX1_HUMAN] | 5 |
| P30040 | Endoplasmic reticulum resident protein 29 OS=Homo sapiens GN=ERP29 PE=1 SV=4 - [ERP29_HUMAN] | 5 |
| P30048 | Thioredoxin-dependent peroxide reductase, mitochondrial OS=Homo sapiens GN=PRDX3 PE=1 SV=3 - [PRDX3_HUMAN] | 5 |
| P39656 | Dolichyl-diphosphooligosaccharide--protein glycosyltransferase 48 kDa subunit OS=Homo sapiens GN=DDOST PE=1 SV=4 - [OST48_HUMAN] | 5 |
| Q15631 | Translin OS=Homo sapiens GN=TSN PE=1 SV=1 - [TSN_HUMAN] | 5 |
| P21796 | Voltage-dependent anion-selective channel protein 1 OS=Homo sapiens GN=VDAC1 PE=1 SV=2 - [VDAC1_HUMAN] | 5 |
| P62993 | Growth factor receptor-bound protein 2 OS=Homo sapiens GN=GRB2 PE=1 SV=1 - [GRB2_HUMAN] | 5 |
| P84095 | Rho-related GTP-binding protein RhoG OS=Homo sapiens GN=RHOG PE=1 SV=1 - [RHOG_HUMAN] | 5 |
| O43490 | Prominin-1 OS=Homo sapiens GN=PROM1 PE=1 SV=1 - [PROM1_HUMAN] | 5 |
| P07741 | Adenine phosphoribosyltransferase OS=Homo sapiens GN=APRT PE=1 SV=2 - [APT_HUMAN] | 5 |
| P08567 | Pleckstrin OS=Homo sapiens GN=PLEK PE=1 SV=3 - [PLEK_HUMAN] | 5 |
| P35237 | Serpin B6 OS=Homo sapiens GN=SERPINB6 PE=1 SV=3 - [SPB6_HUMAN] | 5 |
| P54920 | Alpha-soluble NSF attachment protein OS=Homo sapiens GN=NAPA PE=1 SV=3 - [SNAA_HUMAN] | 5 |
| Q5TFQ8 | Signal-regulatory protein beta-1 isoform 3 OS=Homo sapiens GN=SIRPB1 PE=1 SV=1 - [SIRBL_HUMAN] | 5 |
| P19878 | Neutrophil cytosol factor 2 OS=Homo sapiens GN=NCF2 PE=1 SV=2 - [NCF2_HUMAN] | 4 |
| P49755 | Transmembrane emp24 domain-containing protein 10 OS=Homo sapiens GN=TMED10 PE=1 SV=2 - [TMEDA_HUMAN] | 4 |
| Q14019 | Coactosin-like protein OS=Homo sapiens GN=COTL1 PE=1 SV=3 - [COTL1_HUMAN] | 4 |
| Q96C19 | EF-hand domain-containing protein D2 OS=Homo sapiens GN=EFHD2 PE=1 SV=1 - [EFHD2_HUMAN] | 4 |
| P30086 | Phosphatidylethanolamine-binding protein 1 OS=Homo sapiens GN=PEBP1 PE=1 SV=3 - [PEBP1_HUMAN] | 4 |
| O00148 | ATP-dependent RNA helicase DDX39A OS=Homo sapiens GN=DDX39A PE=1 SV=2 - [DX39A_HUMAN] | 4 |
| P04632 | Calpain small subunit 1 OS=Homo sapiens GN=CAPNS1 PE=1 SV=1 - [CPNS1_HUMAN] | 4 |
| P07858 | Cathepsin B OS=Homo sapiens GN=CTSB PE=1 SV=3 - [CATB_HUMAN] | 4 |
| P36871 | Phosphoglucomutase-1 OS=Homo sapiens GN=PGM1 PE=1 SV=3 - [PGM1_HUMAN] | 4 |
| P61225 | Ras-related protein Rap-2b OS=Homo sapiens GN=RAP2B PE=1 SV=1 - [RAP2B_HUMAN] | 4 |
| Q5JTV8 | Torsin-1A-interacting protein 1 OS=Homo sapiens GN=TOR1AIP1 PE=1 SV=2 - [TOIP1_HUMAN] | 4 |
| P00492 | Hypoxanthine-guanine phosphoribosyltransferase OS=Homo sapiens GN=HPRT1 PE=1 SV=2 - [HPRT_HUMAN] | 4 |
| P62873 | Guanine nucleotide-binding protein G(I)/G(S)/G(T) subunit beta-1 OS=Homo sapiens GN=GNB1 PE=1 SV=3 - [GBB1_HUMAN] | 4 |
| O60664 | Perilipin-3 OS=Homo sapiens GN=PLIN3 PE=1 SV=3 - [PLIN3_HUMAN] | 4 |
| P07203 | Glutathione peroxidase 1 OS=Homo sapiens GN=GPX1 PE=1 SV=4 - [GPX1_HUMAN] | 4 |
| P17858 | 6-phosphofructokinase, liver type OS=Homo sapiens GN=PFKL PE=1 SV=6 - [K6PL_HUMAN] | 4 |
| P39687 | Acidic leucine-rich nuclear phosphoprotein 32 family member A OS=Homo sapiens GN=ANP32A PE=1 SV=1 - [AN32A_HUMAN] | 4 |
| P49721 | Proteasome subunit beta type-2 OS=Homo sapiens GN=PSMB2 PE=1 SV=1 - [PSB2_HUMAN] | 4 |
| P51572 | B-cell receptor-associated protein 31 OS=Homo sapiens GN=BCAP31 PE=1 SV=3 - [BAP31_HUMAN] | 4 |
| P60842 | Eukaryotic initiation factor 4A-I OS=Homo sapiens GN=EIF4A1 PE=1 SV=1 - [IF4A1_HUMAN] | 4 |
| P61163 | Alpha-centractin OS=Homo sapiens GN=ACTR1A PE=1 SV=1 - [ACTZ_HUMAN] | 4 |
| P62942 | Peptidyl-prolyl cis-trans isomerase FKBP1A OS=Homo sapiens GN=FKBP1A PE=1 SV=2 - [FKB1A_HUMAN] | 4 |
| Q15691 | Microtubule-associated protein RP/EB family member 1 OS=Homo sapiens GN=MAPRE1 PE=1 SV=3 - [MARE1_HUMAN] | 4 |
| Q92688 | Acidic leucine-rich nuclear phosphoprotein 32 family member B OS=Homo sapiens GN=ANP32B PE=1 SV=1 - [AN32B_HUMAN] | 4 |
| Q96KP4 | Cytosolic non-specific dipeptidase OS=Homo sapiens GN=CNDP2 PE=1 SV=2 - [CNDP2_HUMAN] | 4 |
| Q9NUV9 | GTPase IMAP family member 4 OS=Homo sapiens GN=GIMAP4 PE=1 SV=1 - [GIMA4_HUMAN] | 4 |
| Q9Y277 | Voltage-dependent anion-selective channel protein 3 OS=Homo sapiens GN=VDAC3 PE=1 SV=1 - [VDAC3_HUMAN] | 4 |
| P59665 | Neutrophil defensin 1 OS=Homo sapiens GN=DEFA1 PE=1 SV=1 - [DEF1_HUMAN] | 4 |
| Q14974 | Importin subunit beta-1 OS=Homo sapiens GN=KPNB1 PE=1 SV=2 - [IMB1_HUMAN] | 4 |
| P01903 | HLA class II histocompatibility antigen, DR alpha chain OS=Homo sapiens GN=HLA-DRA PE=1 SV=1 - [DRA_HUMAN] | 3 |
| Q9BRF8 | Calcineurin-like phosphoesterase domain-containing protein 1 OS=Homo sapiens GN=CPPED1 PE=1 SV=3 - [CPPED_HUMAN] | 3 |
| O60234 | Glia maturation factor gamma OS=Homo sapiens GN=GMFG PE=1 SV=1 - [GMFG_HUMAN] | 3 |
| P00390 | Glutathione reductase, mitochondrial OS=Homo sapiens GN=GSR PE=1 SV=2 - [GSHR_HUMAN] | 3 |
| O95436 | Sodium-dependent phosphate transport protein 2B OS=Homo sapiens GN=SLC34A2 PE=1 SV=3 - [NPT2B_HUMAN] | 3 |
| P04792 | Heat shock protein beta-1 OS=Homo sapiens GN=HSPB1 PE=1 SV=2 - [HSPB1_HUMAN] | 3 |
| P07108 | Acyl-CoA-binding protein OS=Homo sapiens GN=DBI PE=1 SV=2 - [ACBP_HUMAN] | 3 |
| P14314 | Glucosidase 2 subunit beta OS=Homo sapiens GN=PRKCSH PE=1 SV=2 - [GLU2B_HUMAN] | 3 |
| P25774 | Cathepsin S OS=Homo sapiens GN=CTSS PE=1 SV=3 - [CATS_HUMAN] | 3 |
| P30050 | 60S ribosomal protein L12 OS=Homo sapiens GN=RPL12 PE=1 SV=1 - [RL12_HUMAN] | 3 |
| P61604 | 10 kDa heat shock protein, mitochondrial OS=Homo sapiens GN=HSPE1 PE=1 SV=2 - [CH10_HUMAN] | 3 |
| Q07020 | 60S ribosomal protein L18 OS=Homo sapiens GN=RPL18 PE=1 SV=2 - [RL18_HUMAN] | 3 |
| Q12905 | Interleukin enhancer-binding factor 2 OS=Homo sapiens GN=ILF2 PE=1 SV=2 - [ILF2_HUMAN] | 3 |
| Q32P51 | Heterogeneous nuclear ribonucleoprotein A1-like 2 OS=Homo sapiens GN=HNRNPA1L2 PE=2 SV=2 - [RA1L2_HUMAN] | 3 |
| Q8IX19 | Mast cell-expressed membrane protein 1 OS=Homo sapiens GN=MCEMP1 PE=1 SV=1 - [MCEM1_HUMAN] | 3 |
| P41222 | Prostaglandin-H2 D-isomerase OS=Homo sapiens GN=PTGDS PE=1 SV=1 - [PTGDS_HUMAN] | 3 |
| Q06033 | Inter-alpha-trypsin inhibitor heavy chain H3 OS=Homo sapiens GN=ITIH3 PE=1 SV=2 - [ITIH3_HUMAN] | 3 |
| O00560 | Syntenin-1 OS=Homo sapiens GN=SDCBP PE=1 SV=1 - [SDCB1_HUMAN] | 3 |
| O75955 | Flotillin-1 OS=Homo sapiens GN=FLOT1 PE=1 SV=3 - [FLOT1_HUMAN] | 3 |
| P61088 | Ubiquitin-conjugating enzyme E2 N OS=Homo sapiens GN=UBE2N PE=1 SV=1 - [UBE2N_HUMAN] | 3 |
| Q14254 | Flotillin-2 OS=Homo sapiens GN=FLOT2 PE=1 SV=2 - [FLOT2_HUMAN] | 3 |
| P16930 | Fumarylacetoacetase OS=Homo sapiens GN=FAH PE=1 SV=2 - [FAAA_HUMAN] | 2 |
| P28072 | Proteasome subunit beta type-6 OS=Homo sapiens GN=PSMB6 PE=1 SV=4 - [PSB6_HUMAN] | 2 |
| P53634 | Dipeptidyl peptidase 1 OS=Homo sapiens GN=CTSC PE=1 SV=2 - [CATC_HUMAN] | 2 |
| P01040 | Cystatin-A OS=Homo sapiens GN=CSTA PE=1 SV=1 - [CYTA_HUMAN] | 2 |
| P07358 | Complement component C8 beta chain OS=Homo sapiens GN=C8B PE=1 SV=3 - [CO8B_HUMAN] | 2 |
